# Dysregulation of SOX17/NRF2 axis confers chemoradiotherapy resistance and emerges as a novel therapeutic target in esophageal squamous cell carcinoma

**DOI:** 10.1186/s12929-022-00873-4

**Published:** 2022-10-30

**Authors:** Chih-Hsiung Hsieh, Wen-Hui Kuan, Wei-Lun Chang, I-Ying Kuo, Hsun Liu, Dar-Bin Shieh, Hsuan Liu, Bertrand Tan, Yi-Ching Wang

**Affiliations:** 1 Department of Pharmacology, College of Medicine, National Cheng Kung University, Tainan, 70101 Taiwan; 2 Department of Internal Medicine, College of Medicine, National Cheng Kung University Hospital, National Cheng Kung University, Tainan, 70101 Taiwan; 3 Department of Biotechnology, College of Life Science, Kaohsiung Medical University, Kaohsiung, 80708 Taiwan; 4 Institute of Oral Medicine, College of Medicine, National Cheng Kung University, Tainan, 70101 Taiwan; 5 Department of Biochemistry, Chang Gung University, Taoyuan, 33302 Taiwan; 6 Institute of Basic Medicine, College of Medicine, National Cheng Kung University, Tainan, 70101 Taiwan; 7 Department of Pharmacology and Institute of Basic Medical Sciences, College of Medicine, National Cheng Kung University, No. 1, University Road, Tainan, 70101 Taiwan, ROC

**Keywords:** Chemoradiotherapy resistance, NRF2, SOX17, DNA methyltransferases, And esophageal squamous cell carcinoma

## Abstract

**Background:**

Esophageal squamous cell carcinoma (ESCC) is the sixth leading cause of cancer-associated death worldwide with a dismal overall 5-year survival rate of less than 20%. The standard first-line therapy for advanced ESCC is concomitant chemo-radiation therapy (CCRT); however, patients usually develop resistance, resulting in unfavorable outcomes. Therefore, it is urgent to identify the mechanisms underlying CCRT resistance and develop effective treatment strategies.

**Methods:**

Patients’ endoscopic biopsy tumor tissues obtained before CCRT treatment were used to perform RNA-seq and GSEA analysis. Immunohistochemical (IHC) staining, chromatin immunoprecipitation (ChIP), and promoter reporter analyses were conducted to investigate the relationship between SOX17 and NRF2. Xenograft mouse models were used to study the role of SOX17/NRF2 axis in tumor growth and the efficacy of carboxymethyl cellulose-coated zero-valent-iron (ZVI@CMC).

**Results:**

In this study, a notable gene expression signature associated with NRF2 activation was observed in the poor CCRT responders. Further, IHC staining of endoscopic biopsy of 164 ESCC patients revealed an inverse correlation between NRF2 and SOX17, a tumor-suppressive transcription factor with low expression in ESCC due to promoter hypermethylation. Using ChIP and promoter reporter analyses, we demonstrated that SOX17 was a novel upstream transcriptional suppressor of NRF2. In particular, SOX17^low^/NRF2^high^ nuclear level significantly correlated with poor CCRT response and poor survival, indicating that the dysregulation of SOX17/NRF2 axis played a pivotal role in CCRT resistance and tumor progression. Notably, the in-house developed nanoparticle ZVI@CMC functioned as an inhibitor of DNA methyltransferases to restore expression of SOX17 that downregulated NRF2, thereby overcoming the resistance in ESCC. Additionally, the combination of ZVI@CMC with radiation treatment significantly augmented anticancer efficacy to inhibit tumor growth in CCRT resistant cancer.

**Conclusion:**

This study identifies a novel SOX17^low^/NRF2^high^ signature in ESCC patients with poor prognosis, recognizes SOX17 as a transcriptional repressor of NRF2, and provides a promising strategy targeting SOX17/NRF2 axis to overcome resistance.

**Supplementary Information:**

The online version contains supplementary material available at 10.1186/s12929-022-00873-4.

## Background

Esophageal cancer ranked the seventh most common type of cancer and the sixth leading cause of cancer-associated death worldwide [1]. Histologically, esophageal squamous cell carcinoma (ESCC) is the predominant types of esophageal cancers and accounts for more than 80% cases [2, 3]. Early surgical resection is so far recognized as the best approach to improve patients’ survival. Unfortunately, most ESCC patients are diagnosed at an advanced disease stage, in which the overall 5-year survival rate is less than 5% [4], and concomitant chemo-radiation therapy (CCRT) is usually recommended as the most appropriate option for the first-line treatment. However, many ESCC patients had limited response to CCRT treatment because their tumor tissues had developed resistance-associated signatures before the therapy, and thus leading to treatment failure and poor outcomes [5, 6]. Therefore, there is an urgent need to find the acquired resistance signature and develop molecular biomarkers for accurately predicting patient’s response to CCRT and helping physicians to choose the best therapeutic modality for each individual patient.

Nuclear factor-erythroid 2-related factor 2 (NRF2, encoded by *NFE2L2*) is a pivot transcription factor mediating cellular redox homeostasis. It is well-known that NRF2 regulates the expression of numerous antioxidant enzymes including aldo–keto reductase (AKR) protein family [7] and NAD(P)H quinone oxidoreductase 1 (NQO1) [8]. NRF2 can also maintain intracellular glutathione homeostasis *via* regulating the expression of glutathione peroxidase 2 (GPX2) [9]. In fact, under unstressed conditions, basal intracellular NRF2 level is very low in normal cells [10]. The activation of NRF2 can protect cells from oxidative and inflammatory stress as well as toxic substances [11]. Nevertheless, overactivation or accumulation of NRF2 confers selective growth advantages to cancer cells [12]. The persistent NRF2 activation in cancer cells (known as NRF2 addiction [13]) promotes cancer cell proliferation by metabolic reprogramming [14], enhances self-renewal of cancer stem cells [15], and represses programmed cell death such as apoptosis [16] and ferroptosis [17]. Thus, whether the gene expression signatures of NRF2 activation is positively associated with CCRT resistance in ESCC patients is worth to be investigated.

NRF2 protein expression is known to be regulated by the E3-ligases KEAP1 [18] or β-TrCP [19]; however, the dysfunction of the two E3-ligases were not common in ESCC, and no significant correlation was found between CCRT response and the expressions of both KEAP1 and β-TrCP [20, 21]. On the other hand, transcriptional activation of *NFE2L2* gene also contributes to NRF2 hyperactivation. For example, oncogenic K-Ras, B-Raf, and Myc were shown to augment the transcription of *NFE2L2* during tumorigenesis [22]. Besides, the activation of PI3K-Akt pathway promotes the nuclear accumulation of NRF2 to reinforce the metabolic reprogramming of cancer cells [14]. However, never has a transcriptional suppressor of *NFE2L2* gene been explored and elucidated. Thus, it is important to identify negative transcriptional regulators of *NEF2L2* gene for better understanding the cause of NRF2 hyperactivation and its relationship with CCRT resistance in ESCC.

Previously, we discovered that the expression of SOX17, a tumor-suppressive transcriptional regulator with a high mobility group (HMG) box domain that interacts with SRY binding site in DNA, is significantly downregulated due to promoter hypermethylation in ESCC patients with poor prognosis [23]. In addition, SOX17 overexpression can transcriptionally inactivate DNA repair and DNA damage responsive genes to enhance the CCRT sensitivity of ESCC [24]. Therefore, to develop an effective strategy for overcoming CCRT resistance, it is critical to elucidate the mechanism by which SOX17 dysregulation leads to CCRT resistance in ESCC patients.

In this study, we discovered that SOX17 functioned as a novel upstream transcriptional repressor of NRF2, and the analyses of clinical specimens showed that SOX17^low^/NRF2^high^ nuclear level was significantly associated with poor CCRT response and poor survival. Interestingly, the carboxymethyl cellulose-coated zero-valent-iron (ZVI@CMC) nanoparticles could dramatically inhibit DNA methyltransferases (DNMTs) to restore SOX17 protein expression and overcome resistance in NRF2^high^ ESCC cells. Our findings not only provide compelling evidence of the SOX17/NRF2 dysregulation in relation to CCRT resistance of ESCC, but also provide insights that help in the development of SOX17/NRF2-targeting treatment to overcome CCRT resistance.

## Materials and methods

### Cell lines and culture conditions

Taiwanese ESCC cell line CE48T was obtained from Dr. Han-Suei Hsu at Division of Thoracic Surgery, Taipei Veterans General Hospital. Japanese ESCC cell line KYSE510 was purchased from the DSMZ-German Collection of Microorganisms and Cell Cultures (Braunschweig, Germany). Japanese ESCC cell line TE2 was obtained from Dr. Muh-Hwa Yang at Division of Medical Oncology, Department of Oncology, Taipei Veterans General Hospital. Normal human esophageal squamous cell line HET-1A was purchased from the American Type Culture Collection. KYSE510 was maintained in RPMI 1640 medium (Gibco, Grand Island, NY, USA). CE48T and TE2 were maintained in DMEM medium (Gibco). Both RPMI 1640 and DMEM media were supplemented with 10% Fetal Bovine Serum (FBS; Gibco) and 1% penicillin/streptomycin (Gibco). HET-1A was maintained in bronchial epithelial basal medium (BEBM; Lonza, Walkersville, MD, USA). All cells were cultured at 37 °C with 5% CO_2_.

To establish the CCRT-resistant cell line, KYSE510, CE48T, and TE2 cells were treated with 2 μM cisplatin, exposed to 5 Gy radiation, and then recovered for cell growth. When the cells reached 50% confluency, they were again treated with 2 μM cisplatin and 5 Gy radiation until receiving a total cumulative dose of 70 Gy radiation. The corresponding parental cells, named as KYSE510-P, CE48T-P, and TE2-P, while the resistant cells were named as KYSE510-R, CE48T-R, and TE2-R.

### Clinical samples of ESCC patients

A total of 164 patients were recruited for this study with appropriate institutional review board permission and informed consent from the patients. Endoscopy tumor biopsy and corresponding normal biopsy samples were collected. Patient’s response to CCRT was evaluated by endoscopic ultrasound (EUS) and computed tomographic (CT) scans after completion of 36 Gy radiotherapy. The evaluation was carried out before and after CCRT therapies at clinics. Good CCRT responders were patients with post-CCRT esophageal wall thickness < 8 mm by EUS and without enlargement or newly developed distant metastatic foci on CT scan, while poor CCRT responders were patients with post-CCRT esophageal wall thickness ≥ 8 mm or with enlargement or newly developed distant metastatic foci on CT scan [25]. The clinical information of patients whose tumor tissues were used for RNA-seq analysis is provided in Additional file 1: Table S1.

### The Cancer Genome Atlas (TCGA) database analysis

TCGA gene expression data for 173 esophageal carcinoma samples were downloaded from the GDC Data Portal (https://portal.gdc.cancer.gov), which included 82 ESCC samples with HTseq-Counts file. The RNA-seq data of the 82 ESCC samples was extracted and analyzed in this study. The HTseq-Counts were converted to FPKM (Fragments Per Kilobase per Million mapped reads) normalized data, and the FPKM data of *SOX17*, *NFE2L2*, and NRF2-regulated genes are provided in Additional file 1: Table S2. The Log2 transformation of FPKM + 1 was used to present the differential expression of these genes.

### RNA sequencing and GSEA analyses

The RNA libraries were prepared by Agilent SureSelect Strand-Specific RNA Library Preparation Kit (Agilent Technologies, Palo Alto, CA, USA). After assessment using the Agilent 2100 Bioanalyzer instrument with the Agilent High Sensitivity DNA Kit (Agilent Technologies), equal amount of the purified RNA libraries was pooled in molecular ratio and then sequenced using Illumina NextSeq 500 platform. The data analysis was performed as described in our previous report [26]. Following, we applied the RNA-seq data to perform gene set enrichment analysis (GSEA) using WikiPathways pathway database [27].

### Expression vectors, promoter constructs, and transfection

The plasmids used in the study are listed in Additional file 1: Table S3. The SOX17 expression construct was kindly provided by Dr. Stephen B. Baylin at Division of Cancer Biology, Sidney Kimmel Comprehensive Cancer Center, Johns Hopkins University. For the SOX17-WT expression vector, the entire coding region of SOX17 cDNA was subcloned in frame into the pcDNA3.1/V5-His B vector (Invitrogen, Waltham, MA, USA). For the SOX17-ΔHMG expression vector, the DNA-binding domain HMG was deleted. The NRF2 expression vector was purchased from Sino Biological (Wayne, PA, USA). The *NFE2L2* promoter, DNA fragment corresponding to residues − 2000 ~  + 10 containing seven SOX17 binding sites (predicted by PROMO software), was amplified by PCR with the primers listed in Additional file 1: Table S4. The PCR product was restricted by *KpnI* and *XhoI* enzymes and then sub-cloned into the pGL4 basic vector (Promega, Madison, WI, USA) to obtain pGL4-NRF2-Luc promoter plasmid.

### Promoter reporter assay

Cells (1 × 10^4^) were plated in each well of 12-well plates. Then, the cells were co-transfected with 1 μg SOX17 expression vector and 1 μg pGL4-NRF2-Luc promoter plasmid. The dual luciferase reporter assay system (Promega) was used to determine gene promoter activity according to the manufacturer's instructions. Data were represented as the means of ratio of firefly luciferase to Renilla luciferase activity by triplicate experiments.

### RNA extraction and reverse transcription-quantitative polymerase chain reaction (RT-qPCR) assay

Total RNA was extracted using TRIzol reagent (Invitrogen), and purified RNA was reversely transcribed into cDNA using High Capacity cDNA Reverse Transcription Kit (Applied Biosystems, Carlsbad, CA, USA). RT-qPCR was performed with SYBR Green Master Mix (Invitrogen) using the StepOnePlus™ Real-Time PCR system (Applied Biosystems). Expression levels were normalized with internal control *β-actin*. The primer sequences are listed in the Additional file 1: Table S4.

### Chromatin immunoprecipitation assay (ChIP assay)

Cells were fixed with 1% formaldehyde and then quenched with glycine, followed by preparation of nuclear lysates using Magna ChIP™ protein G Kit (Millipore, Burlington, MA, USA). Nuclear lysates were sonicated to obtain DNA fragments around 500 bp and then subjected to immunoprecipitation for 16 h at 4 °C using 4 µg anti-SOX17 antibody (R&D Systems, Minneapolis, MN, USA) or normal IgG (negative control). The levels of targeted genes in ChIP products were determined by RT-qPCR. Primers are listed in Additional file 1: Table S4.

### Protein extraction and Western blotting

Cells were lysed on ice using the RIPA buffer containing protease inhibitors cocktail (Sigma-Aldrich, Saint Louis, MO, USA). Lysates were then centrifuged at 13,200 rpm for 15 min. Equal amount (50 µg) of protein extract was separated on the 8% SDS-PAGE and transferred onto a polyvinyl difluoride (PVDF) membrane. The membranes were blocked with 5% skim milk in Tris-buffered saline with 0.1% Tween-20 for 1 h at room temperature and subsequently incubated overnight with primary antibodies, followed by incubation with horseradish peroxidase-conjugated secondary antibodies. The antibody conditions are described in Additional file 1: Table S5.

### Immunohistochemistry (IHC) staining

All slides were dewaxed with xylene/ethanol, and antigen was retrieved with TRS buffer (pH 9.0) at 100 °C for 10 min. The slides were then incubated with primary antibodies. Diaminobenzidine tetrahydroxychloride (DAB) solution was applied to detect peroxidase activity, followed by counterstaining with hematoxylin. Histopathological review and IHC scoring were performed blindly without any knowledge of clinical and pathologic characteristics of the patients. For each slide, the surrounding non-neoplastic stroma served as an internal control. The level of staining intensity and percentage was graded using a five-tier system. The sample was scored as 5 if > 80% tumor cells were positive staining; 4 for 60–80%; 3 for 40–60%; 2 for 20–40%; 1 if < 20%. As previously defined [24], SOX17 protein level was graded as low expression if the score of nuclear staining was lower than 60%. According to the criteria used in a recent study [28], NRF2 protein level was graded as high expression if the score of nuclear staining was higher than 20%. The antibodies conditions are listed in the Additional file 1: Table S5.

### Immunofluorescence staining

For immunofluorescence staining, Opal stain kit (PerkinElmer, Waltham, MA, USA) was utilized. After cell fixation with 4% formaldehyde, antigen retrieval was performed with citrate buffer (pH 6.0) at 100 °C for 20 min. After blocking, the slides were incubated with primary antibody of β-TrCP, NRF2 or DNMT1, followed by incubation with secondary antibody polymer HRP and subsequently with Opal fluorophore for 10 min at room temperature. Finally, DAPI was applied for nuclei staining, and images were visualized by a fluorescence microscope (Olympus, Tokyo, Japan). The antibodies conditions are listed in the Additional file 1: Table S5.

### Colony formation assay

After transfection or treatment, cells were seeded at low density (500 cells per well) in 6-well plates for 6–8 days. The cells were washed with PBS twice and fixed at room temperature, followed by staining with crystal violet for 30 min. Cell colonies were counted and analyzed using ImageJ software.

### Transwell invasion assay

The transwell membranes (Falcon, Franklin Lakes, NJ, USA) were pre-coated with Matrigel (Corning, New York, NY, USA) 1 day before the experiment. 5 × 10^5^ cells were seeded onto the upper chamber of transwell with 1 ml serum-free medium, and the lower chamber was filled with 2 ml medium containing 20% FBS, followed by incubation at 37 °C for 20 h. Cells invading to the reverse side of transwell membrane were fixed with 1% formaldehyde and then stained with 0.1% crystal violet at room temperature. Cell images were randomly photographed using Olympus CKX53 and analyzed using ImageJ software.

### Wound healing assay

Cells (5 × 10^4^) were seeded into each compartment of the culture insert (Ibidi, Martinsried, Germany). A cell-free gap of 500 µm was created after removing the insert. At the indicated time points, cell images were randomly photographed using Olympus CKX53, and the cell-free gap was analyzed using ImageJ software.

### Cell viability assay

After ZVI@CMC or radiation treatment, cells were incubated with 0.5 mg/ml 3-(4,5-dimethylthiazol-2-yl)-2,5-diphenyltetrazolium bromide (MTT) for 4 h at 37 °C. Crystals were then dissolved in dimethyl sulfoxide, and the optical absorbance at 570 nm was measured.

### Intracellular ROS measurement

10^5^ cells were seeded into each well of 6-well plates. After ZVI@CMC treatment for 6 h, cells were washed twice with PBS and then incubated with 10 μM H_2_DCFDA (Sigma-Aldrich) at 37 °C for 30 min in the dark. After washing with PBS, the fluorescence intensities were measured by flow cytometry (CytoFLEX™, Beckman coulter, Brea, CA, USA).

### DNA bisulfite conversion and methylation-specific polymerase chain reaction (MSP)

Genomic DNA was extracted using Quick-DNA™ Miniprep Plus Kit (Zymo Research, Orange, CA, USA), and then equal amount of DNA samples was treated with EZ DNA methylation-Gold kit (Zymo Research). The methylation level was determined using the MSP assay with primers specific for either methylated or unmethylated DNA. Primers are listed in Additional file 1: Table S4.

### Animal studies

All animal experiments were performed in compliance with institutional guidelines for use and care of animals. To investigate the SOX17/NRF2 pathway on tumor growth, 5–6-week-old BALB/c nude mice were subcutaneously implanted with 5 × 10^6^ KYSE510-R cells. To evaluate the anti-tumor effects, 5–6-week-old mice were subcutaneously implanted with 5 × 10^6^ KYSE510-P or -R cells for ZVI@CMC single treatment, or the combination of ZVI@CMC and radiation treatment.

When tumor volume reached 100 mm^3^, the mice were intravenously injected with PBS or ZVI@CMC at the doses and times indicated. Mice were weighed, and the volumes of the xenografts were measured and quantified during experiment. Tumor tissues were excised and fixed with 4% formaldehyde (Sigma-Aldrich) at the endpoint of experiments.

### Statistical analysis

The statistical analyses of SOX17 and NRF2 expression level, overall survival, and patients’ CCRT response were performed using Statistical Package for the Social Sciences version 26.0 (SPSS Inc., Chicago, IL, USA). The Chi-square test and multivariate logistic regression analyses were conducted. Correlations were examined using Pearson’s correlation test. Overall survival curves were calculated according to the Kaplan–Meier method by the log-rank test. Three independent experiments for cell studies and six mice per group for animal studies were analyzed. Two-tailed Student’s t-test and one-way ANOVA test were used in cell and animal studies. Data represented mean ± s.e.m. The levels of statistical significance were expressed as *p*-values, *p < 0.05; **p < 0.01; ***p < 0.001; ns: non-significant.

## Results

### NRF2 activation signatures in ESCC cells and endoscopic biopsy tissues revealed resistance against CCRT treatment.

**Fig. 1:**
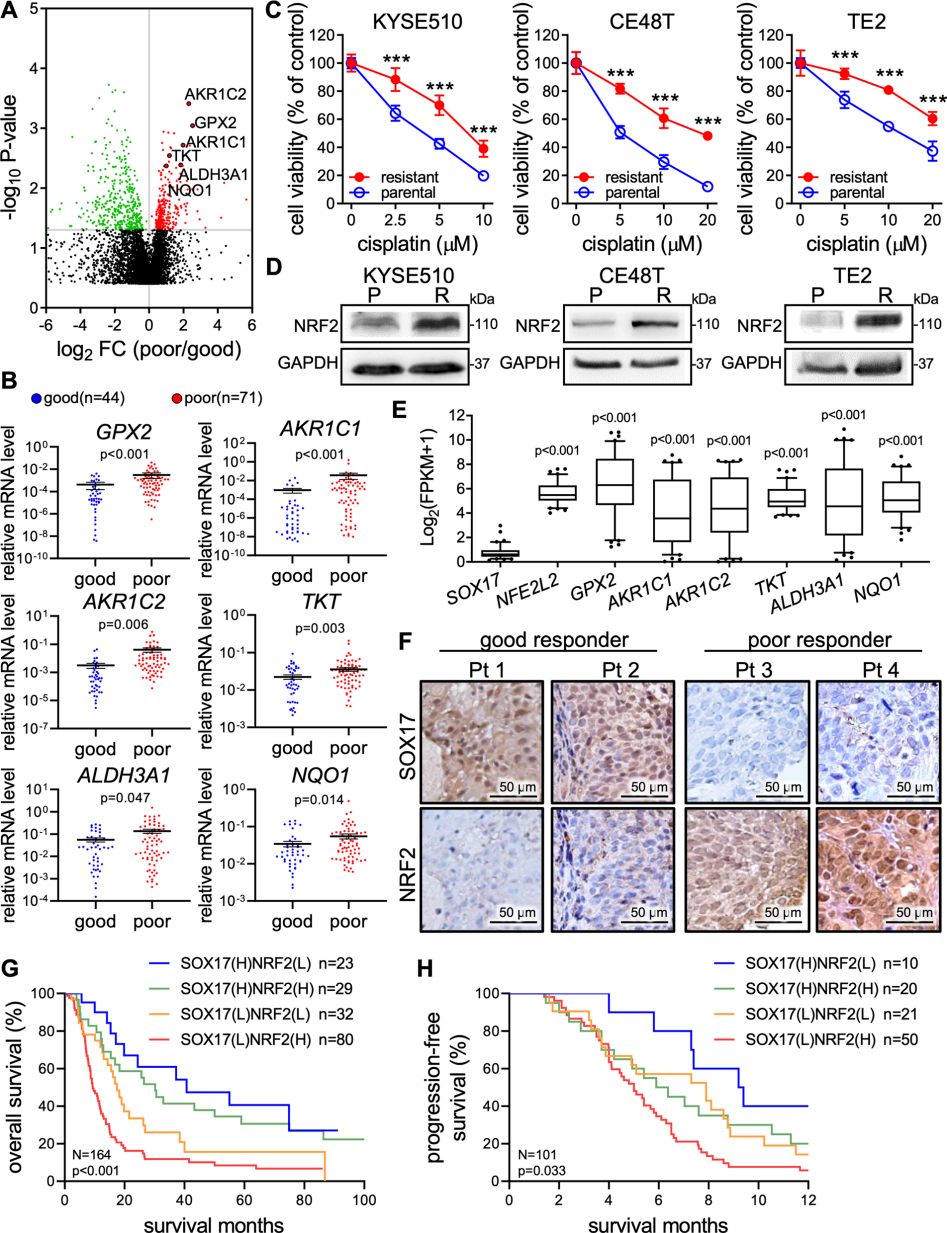
Expression signature analysis of endoscopic biopsy specimens from patients with different CCRT responses. **A** Volcano plot with annotated NRF2 downstream genes that were significantly upregulated in poor CCRT responders (n = 12) compared to good responders (n = 16). **B** Upregulation of NRF2 downstream genes in poor responders was further validated by using qRT-PCR analysis in 44 good plus 71 poor responders. **C** MTT assay of resistant and parental ESCC cells treated with various concentrations of cisplatin for 72 h. **D** Basal levels of NRF2 protein expression in the resistant and parental cells were examined by Western blot analysis. GAPDH was used as an internal control. **E** TCGA database analysis of expression of *SOX17*, *NFE2L2*, and NRF2-regulated genes in ESCC (n = 82). The statistics analysis compared *NFE2L2* and NRF2-regulated genes to *SOX17*. **F** IHC staining showed an inverse correlation between SOX17 and NRF2 protein expression, and SOX17^low^/NRF2^high^ was observed in poor responders. **G** and **H** Kaplan–Meier curves of overall (n = 164) (**G**) and progression-free (n = 101) (**H**) survival analysis based on the status of SOX17 and NRF2 expression in the nucleus. H: high expression; L: low expression. Data represents mean ± s.e.m. ns: non-significant; *p < 0.05; **p < 0.01; ***p < 0.001

To identify gene expression signatures associated with CCRT resistance in ESCC, we performed gene set enrichment analysis (GSEA) of RNA sequencing (RNA-seq) on endoscopic biopsy tissues from patients with good (n = 16) or poor (n = 12) responses to CCRT treatments whose clinical information is shown in Additional file 1: Table S1. The pathway enrichment analysis by GSEA identified the NRF2 pathway as the highest ranked gene signature in the poor responders, and several NRF2-regulated cytoprotective genes were upregulated in ESCC patients with poor responses to CCRT (Fig. 1A; Additional file 1: Fig. S1A–C). To further validate these genes in more patients, we performed RT-qPCR analysis on 115 (n = 44 good responders; n = 71 poor responders) endoscopic biopsy specimens from ESCC patients who received CCRT after the endoscopic biopsy. The RT-qPCR results showed that the expressions of NRF2-regulated *AKR1C1*, *AKR1C2*, *GPX2*, *NQO1*, *ALDH3A1*, and *TKT* were significantly increased in the poor responders (Fig. 1B). This result confirmed the activation of NRF2-mediated cytoprotective program in CCRT resistant tumors and suggested that these genes can be potential biomarkers.

To validate the role of NRF2 in CCRT response, KYSE510, CE48T, and TE2 cells were pretreated with *tert*-butylhydroquinone (tBHQ), a NRF2 activator, before cisplatin treatment and then subjected to MTT assay. We found that cisplatin dose-dependently reduced the cell viability, and the tBHQ pretreatment significantly attenuated the cisplatin-induced viability inhibition (Additional file 1: Fig. S1D), suggesting that NRF2 activation may confer cisplatin resistance in the cancer cells. Further, we established CCRT resistant sublines of these cells. MTT assay showed that under the cisplatin treatment at same concentration, the cell viability of resistant cells was higher than that of parental cells (Fig. 1C). Notably, immunoblotting confirmed that resistant cells expressed more NRF2 proteins than did their corresponding parental cells (Fig. 1D). In addition, NRF2-regulated cytoprotective genes were upregulated in the resistant cells (Additional file 1: Fig. S1E). Taken together, these results suggested that the activation of NRF2 contributes to resistance in ESCC cells.

### Inverse correlation between SOX17 and NRF2 protein expression in ESCC patients

Previously, we reported that SOX17 promoter hypermethylation leads to low expression of its mRNA and protein, which correlates with poor prognosis in ESCC patients [23]. In addition, we demonstrated that overexpression of SOX17 sensitizes CCRT response in ESCC xenograft models [24]. Furthermore, the analysis of a TCGA database (TCGA-ESCA) showed that the mRNA of *SOX17* in ESCC patients was obviously low expressed while those of *NFE2L2* and its downstream genes were highly expressed (Fig. 1E). Therefore, we further examined SOX17 and NRF2 protein expression in the tumor tissues derived from endoscopic biopsy samples of good or poor CCRT responders. Consistently, by examination of immunohistochemistry (IHC) staining at the same tissue region, we found an inverse association of protein expression between the two transcription regulators SOX17 and NRF2 in nucleus (Fig. 1F). Of note, decreased SOX17 expression accompanied with high nuclear expression of NRF2 was observed in poor CCRT responders. In most clinical cases (62.8% patients), the nuclear expression of NRF2 presented an inverse correlation with that of SOX17 (Additional file 1: Fig. S1F and G). Similarly, Chi-Square analysis revealed a significant inverse correlation between SOX17 and NRF2 expression (Table 1). In particular, low SOX17 expression or high NRF2 expression in ESCC patients was significantly associated with poor CCRT responses (Additional file 1: Fig. S1H).

**Table 1:** The nuclear level of SOX17/NRF2 was associated with CCRT response of ESCC patients ^a^

Clinical parameters	Total patients	SOX17		Total patients	NRF2	
		Protein expression			Protein expression	
	164 ^b^	N = 52	N = 112	164 ^b^	N = 55	N = 109
		32.1%	67.9%		33.5%	66.5%
		High	Low		Low	High
**NRF2**						
**Low**	55	23 (41.8)	32 (58.2)^**0.048**^			
**High**	109	29 (26.6)	80 (73.4)			
**Age**						
< 55	67	15 (22.3)	52 (77.7)^**0.033**^	67	22 (32.8)	45 (67.2)^0.874^
≥ 55	97	37 (38.1)	60 (61.9)	97	33 (34.0)	64 (66.0)
**Sex**						
Male	157	48 (30.6)	109 (69.4)^0.174^	157	52 (33.1)	105 (66.9)^0.593^
Female	7	4 (57.1)	3 (42.9)	7	3 (42.9)	4 (57.1)
**Smoker**						
No	22	9 (40.9)	13 (59.1)^0.319^	22	11 (50.0)	11 (50.0)^0.079^
Yes	142	43 (28.2)	99 (71.8)	142	44 (31.0)	98 (69.0)
**Stage**						
I–III	86	32 (37.2)	54 (62.8)^0.112^	86	25 (29.1)	61 (70.9)^0.203^
IV	78	20 (25.6)	58 (74.4)	78	30 (38.5)	48 (61.5)
**T Stage** ^c^						
I–III	129	41 (31.8)	88 (68.2)^0.949^	129	42 (32.6)	87 (67.4)^0.763^
IV	34	11 (32.3)	23 (67.7)	34	12 (35.3)	22 (64.7)
**N Stage** ^c^						
0	19	9 (47.4)	10 (52.6)^0.124^	19	5 (26.3)	14 (73.7)^0.502^
1	144	43 (29.9)	101 (70.1)	144	49 (34.0)	95 (66.0)
**M Stage** ^c^						
0	85	31 (36.5)	54 (63.5)^0.151^	85	25 (29.4)	60 (70.6)^0.266^
1	77	20 (26.0)	57 (74.0)	77	29 (37.7)	48 (62.3)
**Response**						
**Good**	43	21 (48.8)	22 (51.2)^**<0.001**^	43	30 (69.8)	13 (30.2)^**<0.001**^
**Poor**	83	12 (14.5)	71 (85.5)	83	11 (13.3)	72 (86.7)

^a ^The data was analyzed by Pearson χ^2^ test with significant P values in bold

^b ^Clinical information was not available in some sub-groups

^c ^T status: primary tumor; N status: lymph node metastasis; M status: distant metastasis

Next, Kaplan–Meier analysis of overall survival (OS) indicated that patients with SOX17^low^/NRF2^high^ signature had significantly lower OS than did those with other signatures (Fig. 1G). Likewise, patients with SOX17^low^/NRF2^high^ signature had significantly shorter progression-free survival (Fig. 1H). Furthermore, the multivariate Cox regression analysis showed that SOX17^low^/NRF2^high^ was a significant predictor of cancer-related death (Table 2). These results indicated that the SOX17^low^/NRF2^high^ signature was strongly associated with poor CCRT responses and poor survival in ESCC patients.

**Table 2:** Cox regression analysis of risk factors for cancer-related death in ESCC patients

Characteristics	Esophageal squamous cell carcinoma patients			
	Univariate analysis		Multivariate analysis	
	HR ^a^ (95% CI^ b^)	*P*-value^ c^	HR (95% CI)	*P*-value
SOX17 expression^ d^				
SOX17^high^	1		1	
SOX17^low^	2.529 (1.681–3.803)	**< 0.001**	2.767 (1.786–4.288)	**< 0.001**
NRF2 expression^ d^				
NRF2^low^	1		1	
NRF2^high^	1.660 (1.127–2.446)	**0.010**	1.614 (1.076–2.421)	**0.021**
SOX17/NRF2 expression^d^				
SOX17^high^ NRF2^low^	1		1	
SOX17^high^ NRF2^high^	1.369 (0.661–2.883)	0.397	1.403 (0.667–2.952)	0.373
SOX17^low^ NRF2^low^	2.125 (1.043–4.329)	**0.038**	2.516 (1.207–5.245)	**0.014**
SOX17^low^ NRF2^high^	3.738 (1.965–7.110)	**< 0.001**	4.313 (2.218–8.384)	**< 0.001**
Age				
< 55 year-old	1		1	
≥ 55 year-old	1.274 (0.886–1.832)	0.191	1.481 (1.002–2.189)	**0.049**
Sex				
Male	1		1	
Female	0.457 (0.169–1.239)	0.124	0.397 (0.138–1.141)	0.086
Smoker				
No	1		1	
Yes	1.077 (0.645–1.797)	0.777	0.915 (0.518–1.619)	0.762
Stage				
Stage I–III	1		1	
Stage IV	1.088 (0.769–1.540)	0.633	1.150 (0.787–1.680)	0.471
T status^ e^				
Stage I–III	1		1	
Stage IV	1.313 (0.857–2.011)	0.211	1.224 (0.793–1.891)	0.362
N status^ e^				
N0	1		1	
N1	1.072 (0.633–1.817)	0.795	0.652 (0.363–1.170)	0.152
M status^ e^				
M0	1		1	
M1	1.051 (0.741–1.489)	0.782	1.117 (0.765–1.633)	0.566

^a ^HR: Hazard ratio

^b ^CI: Confidence interval

^c^ Bold values indicate statistical significance (P < 0.05)

^d ^SOX17^high^, high expression for SOX17; SOX17^low^, low expression for SOX17; NRF2^high^, high expression for NRF2; NRF2^low^, low expression for NRF2

^e^ T status: primary tumor; N status: lymph node metastasis; M status: distant metastasis

### SOX17 directly binds to the promoter of NRF2 and acts as a transcription repressor

**Fig. 2:**
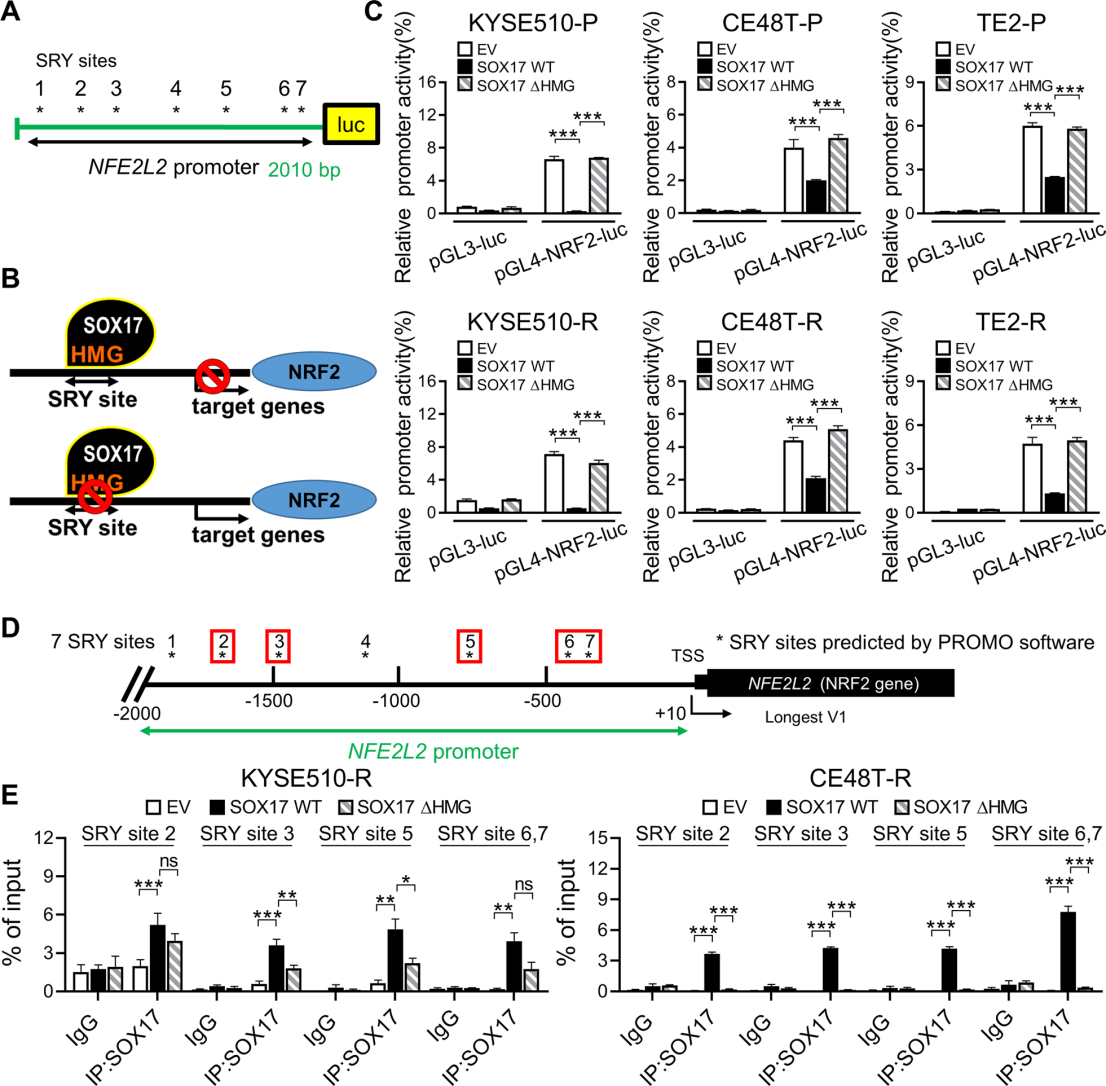
SOX17 functions as a transcriptional repressor of NRF2. **A** Schematic of reporter plasmid construct used in luciferase reporter assay. The *NFE2L2* promoter (− 2000 ~  + 10 bp) containing seven SOX17 binding sites was sub-cloned into the pGL4 basic vector. **B** Schematic of the hypothesis that SOX17-mediated transcriptional repression on NRF2 model. SRY sites: SOX17 binding residues. **C** Dual luciferase promoter reporter assay was performed to examine the effects of SOX17 wild type (SOX17-WT) or HMG box deletion (SOX17-ΔHMG) overexpression on promoter activity of *NFE2L2* in ESCC cells. **D** Promoter map for *NFE2L2* gene. Regions examined with ChIP assay are marked by red circle-backslash symbol. **E** ChIP-qPCR assay was performed to measure SOX17 binding ability to the promoter region of *NFE2L2* in ESCC cells. Data represents mean ± s.e.m. ns: non-significant; *p < 0.05; **p < 0.01; ***p < 0.001

Notably, SOX17 was recognized as a tumor-suppressive transcription factor that negatively regulates DNA repair genes in ESCC [23]. Since the analysis of clinical specimens revealed an inverse correlation between SOX17 and NRF2, we suggested that the downregulated transcription activity of SOX17 in ESCC may cause dysregulation of cytoprotective enzymes controlled by NRF2, resulting in CCRT resistance. Interestingly, seven SOX17 binding sites (sex-determining region Y (SRY) sites) are found on *NFE2L2* promoter by using PROMO software (Fig. 2A); therefore, we hypothesized that SOX17 could be an upstream transcriptional suppressor of NRF2 (Fig. 2B). To investigate whether SOX17 could transcriptionally regulate the expression of *NFE2L2*, a 2010-bp fragment of *NFE2L2* promoter was used to construct a luciferase reporter plasmid and subjected to luciferase reporter assay. Of note, the promoter activities were significantly inhibited by overexpression of wild type SOX17 (SOX17-WT), whereas the overexpression of HMG box-deleted SOX17 (SOX17-ΔHMG) restored the promoter activities in both parental and resistant cells (Fig. 2C).

Next, chromatin immunoprecipitation (ChIP)-qPCR assay was conducted to further validate whether SOX17 directly binds to the SRY sites in *NFE2L2* promoter (Fig. 2D). We found that SOX17-WT could bind to the SRY sites on *NFE2L2* promoter; however, the SOX17-ΔHMG largely reduced binding to *NFE2L2* promoter (Fig. 2E). Furthermore, we found that histone deacetylase 1 (HDAC1) could serve as a corepressor with SOX17 to suppress expression of *NFE2L2* (Additional file 1: Fig. S2A). Together, these results showed that SOX17 directly inhibits the expression of NRF2, and HMG domain of SOX17 for mediating transcriptional regulation is involved in this inhibitory interaction.

### SOX17 downregulates protein expression of NRF2 and mRNA expression of NRF2 targeted genes

**Fig. 3:**
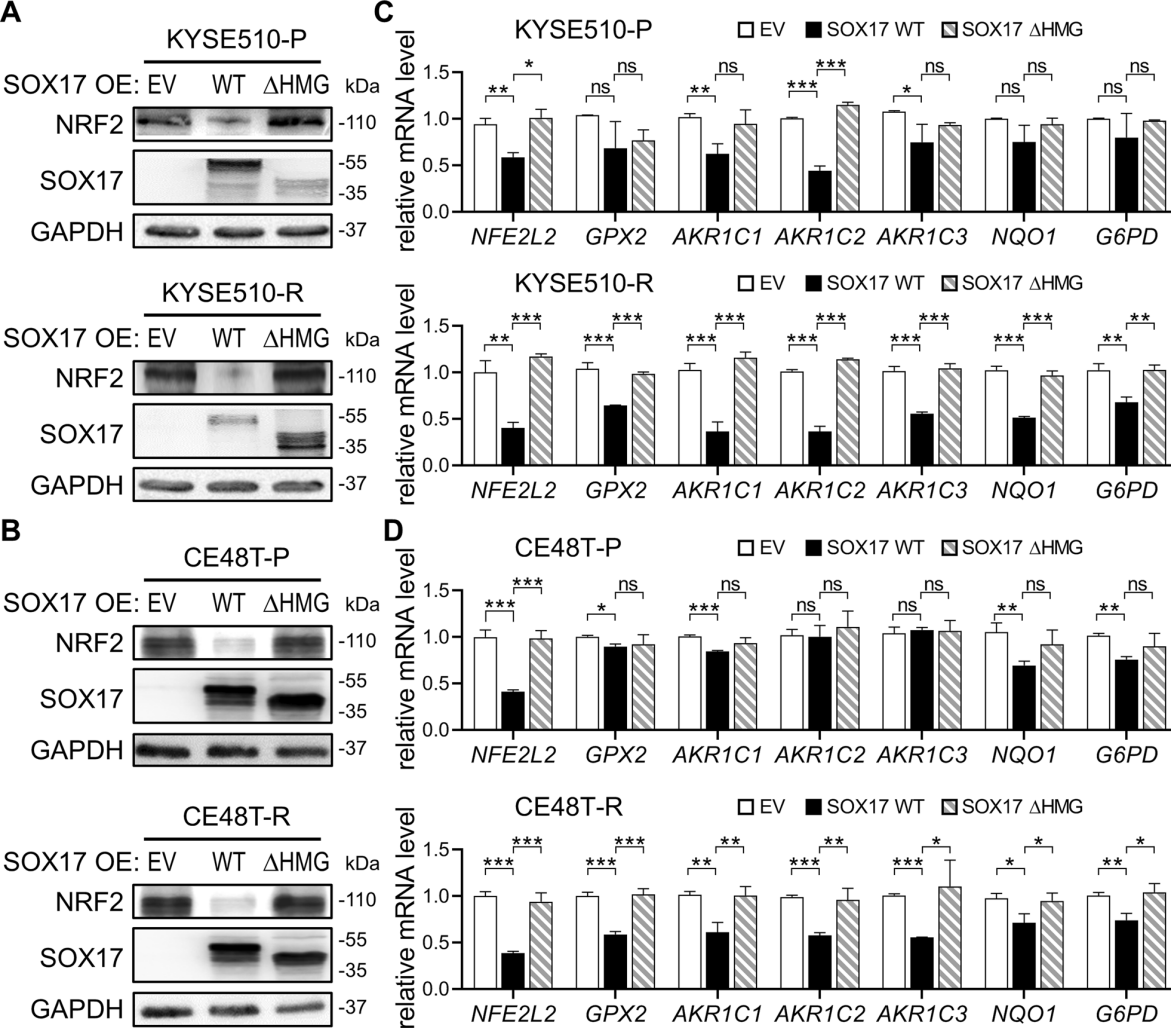
SOX17 overexpression downregulated NRF2 protein expression and the mRNA level of NRF2-targeted genes. **A**, **B** Western blot showed a decreased expression level of NRF2 protein after SOX17-WT overexpression, while no change in NRF2 protein expression after SOX17-ΔHMG overexpression in KYSE510 pair cells (**A**) and in CE48T pair cells (**B**). GAPDH was used as an internal control. **C** and **D,** The mRNA expressions of *NFE2L2* and NRF2-regulated genes *GPX2*, *AKR1C1*, *AKR1C2*, *AKR1C3*, *NQO1*, and *G6PD* were determined by RT-qPCR analysis after SOX17 overexpression for 72 h in KYSE510 pair cells (**C**) and in CE48T pair cells (**D**). *β-actin* was used as an internal control. Data represents mean ± s.e.m. ns: non-significant; *p < 0.05; **p < 0.01; ***p < 0.001

Our data so far demonstrated that SOX17 directly repressed the expression of NRF2 in an HMG domain-dependent manner. Subsequently, we investigated whether SOX17 downregulated the protein level of NRF2 and further reduced the mRNA level of NRF2-targeted genes. IHC staining of the SOX17-overexpressed xenografts showed that NRF2 protein level was remarkably reduced (Additional file 1: Fig. S3A). Consistently, the results of immunoblotting showed that the NRF2 protein expressions in KYSE510, CE48T, and their CCRT-resistant sublines were all decreased after SOX17-WT overexpression, whereas the NRF2 protein levels of SOX17-ΔHMG-overexpressed cells were similar to that of EV control (Fig. 3A and B). In addition, RT-qPCR results demonstrated that mRNA levels of *NFE2L2* and NRF2-regulated cytoprotective genes were also downregulated in the ESCC cells with SOX17-WT overexpression, while the expressions of these genes were not affected after SOX17-ΔHMG overexpression (Fig. 3C and D), indicating that the transcription activity of SOX17 is negatively correlated with the NRF2-regulated cytoprotective pathway. Of note, KYSE510-R and CE48T-R cells were more sensitive to the SOX17-WT-induced downregulation of NRF2-targeted gene expression than their parental cells. Collectively, these results suggest that the downregulated transcription activity of SOX17 in ESCC causes dysregulation of cytoprotective enzymes controlled by NRF2, and thus resulting in CCRT resistance.

### Alterations of cell behavior and tumor growth after overexpression of SOX17 and/or NRF2

**Fig. 4:**
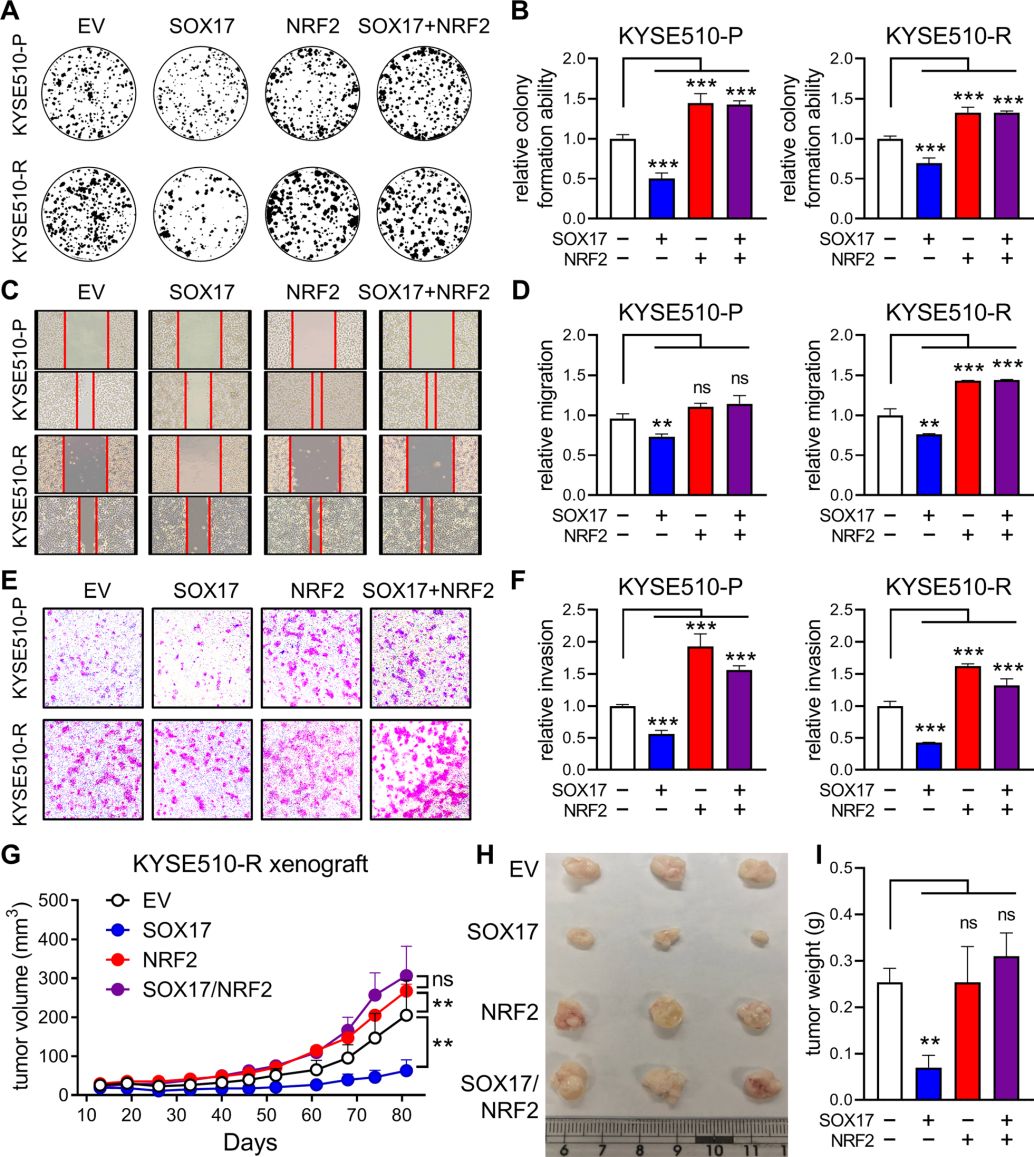
The effects of SOX17 and/or NRF2 overexpression on cell behaviors and tumor growth. **A**, **B** Colony formation assay of KYSE510 pair cells manipulated with SOX17 and/or NRF2 expression. The colonies were stained on day 8 after seeding (**A**), and the colony formation ability was quantified (**B**). **C**, **D** Wound healing assay of KYSE510 pair cells manipulated with SOX17 and/or NRF2 expression. Cells were monitored for their ability to migrate into the wound gap. The wound gap was photographed (**C**) and quantified (**D**) at 10 h. **E**, **F** Transwell invasion assay of KYSE510 pair cells manipulated with SOX17 and/or NRF2 expression. The invaded cells were photographed (**E**) and quantified (**F**) at 20 h. **G** KYSE510-R cells transfected with SOX17 and/or NRF2 were subcutaneously injected into BALB/c nude mice and observed for tumor growth. **H**, **I** Tumor size (**H**) and tumor weight (**I**) were measured at the end of the experiment. Data represents mean ± s.e.m. ns: non-significant; *p < 0.05; **p < 0.01; ***p < 0.001

Next, we verified the effects of SOX17 or NRF2 expression on cell growth, migration, and invasion abilities of ESCC cells. The colony formation abilities of both KYSE510-P and KYSE510-R cells were significantly decreased in the SOX17-overexpressed cells as compared to the control cells (Fig. 4A and B; Additional file 1: Fig. S4A). On the contrary, colony formation ability was enhanced in the NRF2-overexpressed cells as compared to the control cells. Similarly, the migration (Fig. 4C and D; Additional file 1: Fig. S4B-D) and invasion (Fig. 4E and F; Additional file 1: Fig. S4E and F) abilities of ESCC cells were impaired by SOX17 overexpression but promoted by NRF2 overexpression. Notably, no significant difference was observed between cells with NRF2 overexpression and those with SOX17/NRF2 co-overexpression, supporting the notion that NRF2 is a downstream target of SOX17, and SOX17 overexpression-induced inhibition effects were attenuated by NRF2 re-expression.

Further, to investigate the effect of SOX17/NRF2 pathway on tumor growth in vivo, KYSE510-R cells with manipulated protein expression were subcutaneously injected into BALB/c nude mice. Consistent with in vitro cell behavior observations, SOX17 overexpression reduced the tumor growth while NRF2 overexpression promoted tumor growth as compared to the control (Fig. 4G–I; Additional file 1: Fig. S4G). No significant difference in tumor growth was observed between the mice with NRF2 overexpression and those with SOX17/NRF2 co-overexpression, confirming that NRF2 functions downstream of SOX17. Together, these in vitro and in vivo results showed that SOX17 is an upstream transcriptional suppressor of NRF2, and increased NRF2 protein expression could promote aggressive cell behaviors and accelerate tumor growth.

### ZVI@CMC nanoparticle serves as a potential NRF2-targeting strategy through SOX17 re-expression

**Fig. 5:**
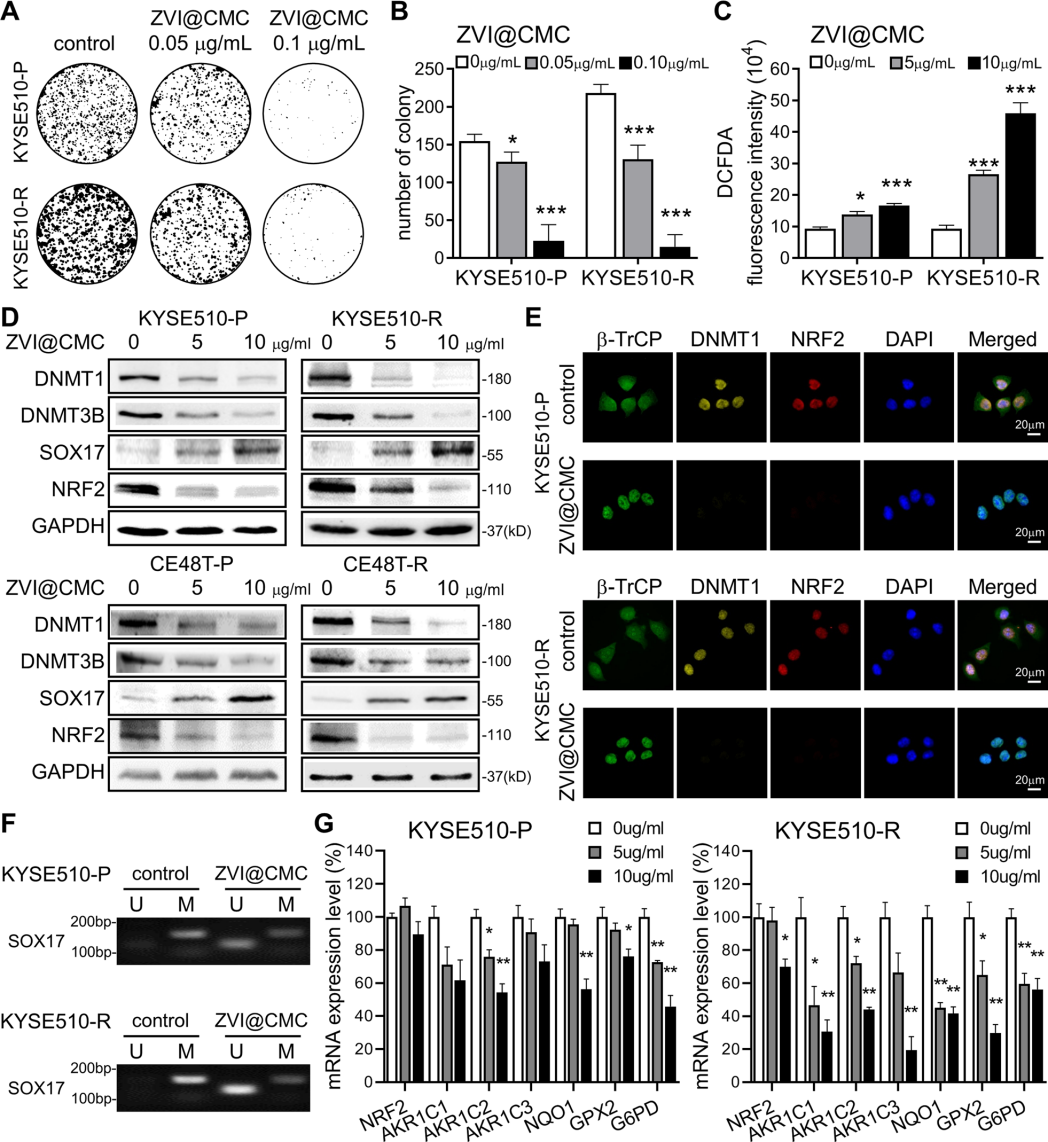
ZVI@CMC nanoparticles elicited DNMT inhibition to restore the expression of SOX17 and NRF2. **A**, **B** Colony formation assay of KYSE510 pair cells treated with ZVI@CMC. The colonies were stained on day 12 after seeding (**A**), and the colony number was quantified (**B**). **C** Intracellular ROS level was determined by flow cytometry analysis of DCFDA fluorescence intensity after ZVI@CMC treatment for 24 h. **D** Immunoblotting of DNMT1, DNMT3B, SOX17, and NRF2 in ESCC cells treated with ZVI@CMC. GAPDH was used as an internal control. **E** Immunofluorescence staining of β-TrCP, DNMT1, NRF2, and DAPI in KYSE510 pair cells treated with ZVI@CMC. **F** Methylation-specific PCR (MSP) demonstrated that ZVI@CMC could reduce the methylation of *SOX17* promoter. M indicates methylated PCR products, and U indicates unmethylated PCR products. **G** RT-qPCR analysis showed that the mRNA expressions of NRF2 downstream genes were downregulated by ZVI@CMC treatment. *β-actin* was used as an internal control. Data represents mean ± s.e.m. ns: non-significant; *p < 0.05; **p < 0.01; ***p < 0.001

Recently, we have demonstrated that ZVI@CMC nanoparticles exhibit anticancer potential by causing ferroptotic cell death in lung cancer and elicit anti-tumor immunity [29]. Interestingly, we found that ZVI@CMC also dose-dependently decreased the survival and proliferation of both KYSE510-P and KYSE510-R cells (Fig. 5A and B; Additional file 1: Fig. S5A). In particular, KYSE510-R cells were more sensitive to the ZVI@CMC-induced cytotoxicity than KYSE510-P. Further, flow cytometry analysis of DCFDA fluorescence intensity showed that ZVI@CMC significantly increased the intracellular reactive oxygen species (ROS) levels (Fig. 5C), and thus causing cancer cell death, evidenced by the MTT result showing that ZVI@CMC-induced cell death was attenuated by the addition of antioxidant vitamin E (Additional file 1: Fig. S5A). Of note, the ZVI@CMC-induced ROS level was more excessive in KYSE510-R than in KYSE510-P cells (Fig. 5C). No apparent difference in viability of normal esophageal epithelial HET-1A cells was observed after ZVI@CMC treatment (Additional file 1: Fig. S5B), indicating that ZVI@CMC exhibited cancer-specific cytotoxicity toward ESCC cells.

We further dissected the molecular mechanism underlying the anticancer effects of ZVI@CMC. As shown in Fig. 5D and Additional file 1: Fig. S5C, ZVI@CMC treatment induced the AMPK/mTOR signaling to enhance p-GSK3/β-TrCP-dependent degradation of NRF2, which agrees with our previous findings [29]. Strikingly, we discovered that ZVI@CMC could also restore the protein expression of SOX17 and reduce NRF2 protein expression (Fig. 5D). Since the low expression of SOX17 in ESCC is attributed to promoter hypermethylation [23], we then examine whether DNA methyltransferases DNMT1 and DNMT3B could be affected by ZVI@CMC treatment. As shown in Fig. 5D and E, the immunofluorescence staining demonstrated that β-TrCP E3-ligase translocated into the nucleus, while the protein levels of DNMT1, DNMT3B, and NRF2 were obviously decreased after ZVI@CMC treatments. Further, the results of methylation-specific PCR (MSP) revealed that ZVI@CMC reduced the methylation level of *SOX17* promoter (Fig. 5F), confirming that the inhibition of DNMTs by β-TrCP-mediated protein degradation partly accounted for the ZVI@CMC-induced restoration of SOX17.

Subsequently, RT-qPCR analysis indicated that NRF2-targeted cytoprotective genes were downregulated after ZVI@CMC treatment (Fig. 5G). Particularly, the expression of these genes was reduced more in KYSE510-R than in KYSE510-P cells. Together, these results suggested that ZVI@CMC dramatically inhibited the NRF2-mediated cytoprotective programs through restoration of SOX17 protein expression by decreasing expression of DNMTs, which may serve as a potential anticancer strategy against ESCC cells both with and without CCRT resistance.

### ZVI@CMC combined with radiation treatment significantly suppressed ESCC tumor growth in vivo

Next, to determine whether ZVI@CMC could exert anti-tumor effect in vivo, xenograft animal models of KYSE510-P and KYSE510-R were established. We found that tumor volume and tumor size were significant reduced after ZVI@CMC treatment as compared to the PBS control (Additional file 1: Fig. S6A and B). Additionally, body weight (Additional file 1: Fig. S6C), blood biochemistry analysis (Additional file 1: Fig. S6D), and H&E-stained tissue sections of major organs (Additional file 1: Fig. S6E) showed no obvious difference between the PBS control and the ZVI@CMC-treated groups.

**Fig. 6:**
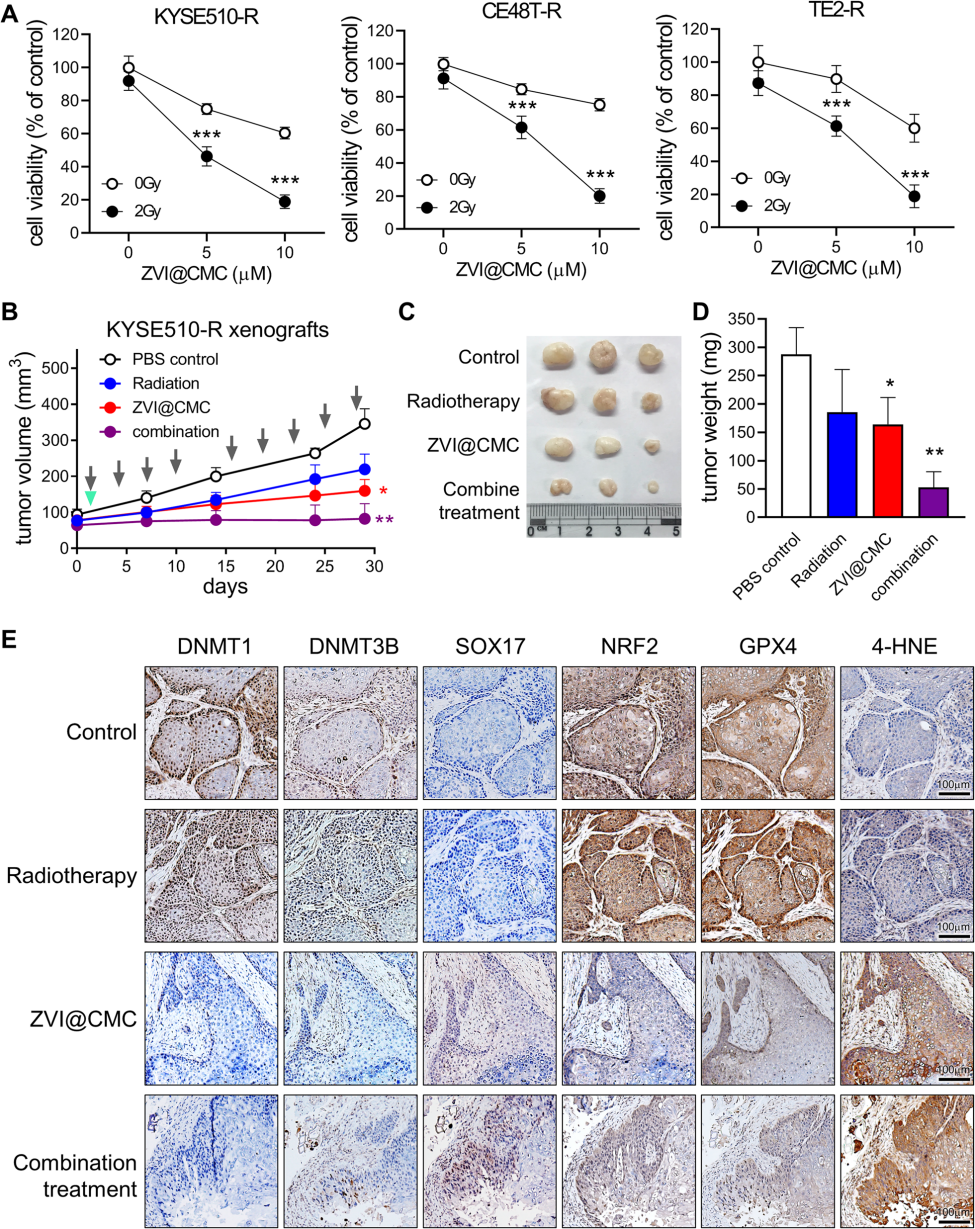
The combination of ZVI@CMC and radiation treatment boosted the anti-tumor efficacy. **A** MTT assay of ESCC resistant cells treated with the combination of ZVI@CMC and radiation treatment for 72 h. **B** Tumor growth of KYSE510-R xenografts treated with ZVI@CMC (indicated as arrow) and/or radiation (indicated as triangle) treatment. **C**, **D** Tumor size (**C**) and tumor weight (**D**) were measured at the end of the experiment. **E** Immunohistochemistry staining revealed the expression of DNMT1, DNMT3B, SOX17, NRF2, GPX4, and 4-HNE in tumor tissues from KYSE510-R xenografts which were treated with ZVI@CMC and/or radiation treatment. Data represents mean ± s.e.m. ns: non-significant; *p < 0.05; **p < 0.01; ***p < 0.001

Based on our previous observation that SOX17 overexpression could sensitize ESCC cells to radiation treatment [24], we further investigated whether ZVI@CMC combined with radiation treatment could augment anti-tumor efficacy and overcome resistance in NRF2^high^ ESCC cells. As shown in Fig. 6A, the cell viabilities of resistant ESCC cells were slightly reduced after exposure to 2 Gy radiation. Interestingly, the combination treatment with ZVI@CMC and radiation significantly decreased the viabilities of ESCC cells both with and without resistance (Fig. 6A; Additional file 1: Fig. S6F).

Further, we examined the in vivo anti-tumor efficacy on KYSE510-R xenografts. The results showed that tumor volume, tumor size, and tumor weight were remarkably reduced after combination treatment with ZVI@CMC and radiation (Fig. 6B-D), while showing no significant body weight loss and change in blood biochemical parameters (Additional file 1: Fig. S6G and H), suggesting the therapeutic potential of this combination treatment to overcome the resistance in ESCC without apparent toxicity. In addition, the IHC staining of xenograft tumor tissue demonstrated that the protein levels of DNMT1 and DNMT3B were significantly decreased after ZVI@CMC treatment (Fig. 6E). Importantly, SOX17 re-expression was observed in the ZVI@CMC-treated tumors while NRF2 protein expression was reduced (Fig. 6E). Furthermore, ZVI@CMC treatment decreased the protein expression of GPX4, a NRF2-regulated anti-ferroptosis protein (Fig. 6E). Besides, the level of 4-HNE, a biomarker of lipid peroxidation and ferroptosis, was dramatically increased after the combination treatment (Fig. 6E). Notably, radiation treatment alone did not affect the expression of DNMT1, DNMT3, SOX17, NRF2, GPX4, and 4-HNE (Fig. 6E). Taken together, these data suggested that ZVI@CMC could sensitize ESCC cells to radiation treatment due to recovery of SOX17/NRF2 axis, and combination treatment could serve as a promising strategy to overcome CCRT resistance in SOX17^low^/NRF2^high^ cancer cells.

## Discussion

**Fig. 7:**
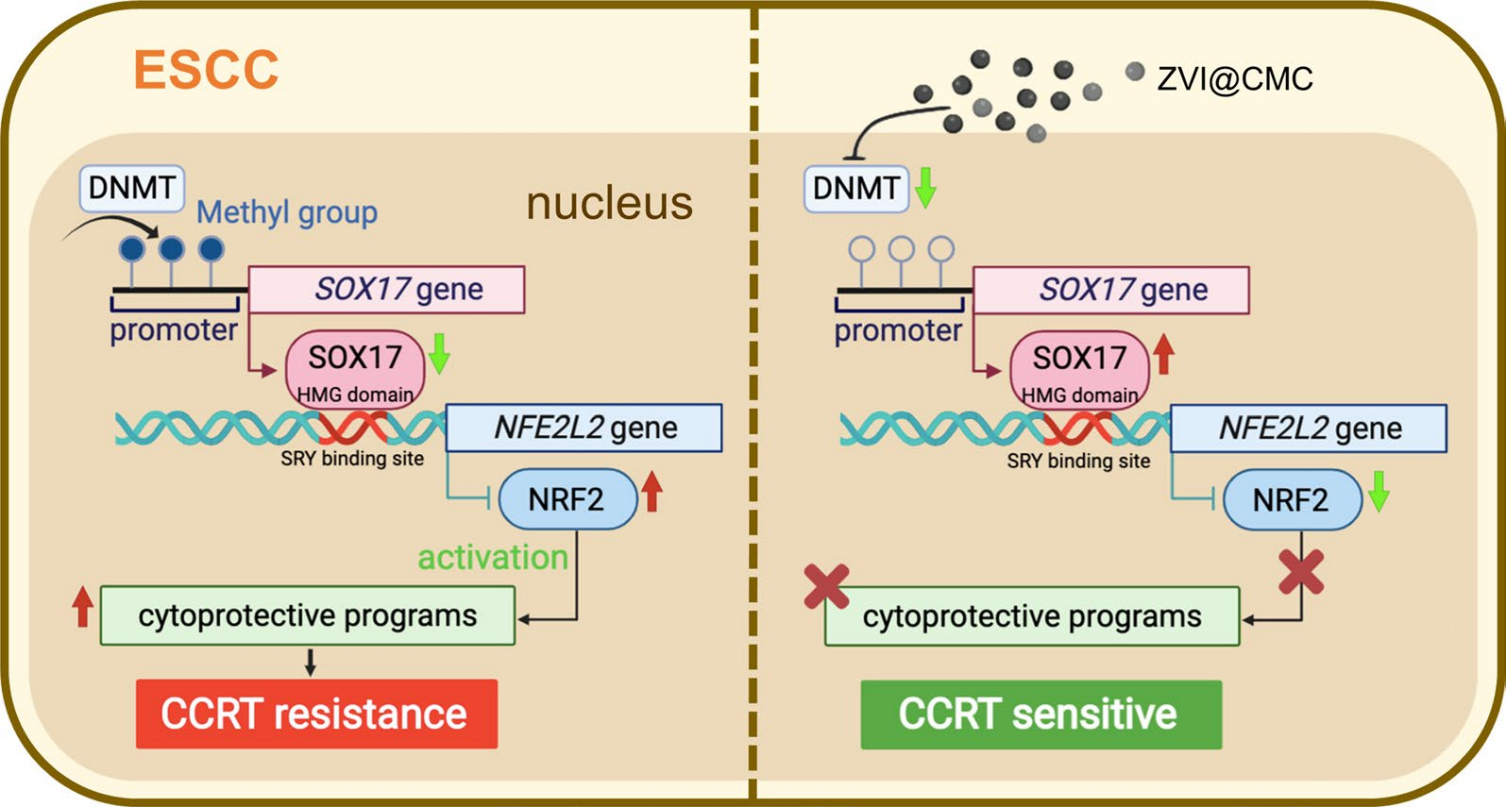
The model of SOX17/NRF2 transcription axis and ZVI@CMC treatment in ESCC. In the CCRT resistant cells, the promoter hypermethylation-induced low SOX17 protein expression causes dysregulation of the cytoprotective enzymes controlled by NRF2, and thus resulting in CCRT resistance (left). Interestingly, ZVI@CMC elicits DNMT inhibition to re-express SOX17 and suppress NRF2-mediated cytoprotective programs in ESCC, and thereby sensitizing cancer cells to CCRT treatment (right)

In this study, we demonstrated a novel link between SOX17 transcriptional deregulation and activation of NRF2-mediated cytoprotective programs in the induction of CCRT resistance in ESCC (Fig. 7). RNA-seq analysis of tissues derived from patient’s endoscopic tumor biopsies revealed that the expressions of several NRF2-targeted antioxidant and detoxification genes were significantly upregulated in poor CCRT responders compared to those in the good responders. IHC of these clinical tissues uncovered an inverse correlation between SOX17 and NRF2. Importantly, results of luciferase assay and ChIP-qPCR analysis provided the first evidence that SOX17 could directly bind to *NFE2L2* promoter to transcriptionally suppress NRF2 expression, which in turn led to downregulation of NRF2-driven cytoprotective program. Interestingly, our innovative ZVI@CMC nanoparticles effectively restored SOX17 protein expression and decreased the expressions of NRF2 as well as NRF2-targeted genes. Moreover, ZVI@CMC treatment combined with radiation treatment elicited remarkable anticancer effects both in vitro and in vivo, suggesting the promising potential of this combination treatment to overcome CCRT resistance in SOX17^low^/NRF2^high^ ESCC cells.

In various types of cancer, aberrant activation of NRF2 is significantly associated with poor clinical outcome of patients [30]. Several oncogenic signaling pathways have been reported to activate NRF2-mediated antioxidant responses. For example, both c-MYC and K-Ras-activating mutations were shown to increase NRF2 expression and promote tumor progression [31, 32]. Additionally, it has been demonstrated that the tumor suppressor P53 can counteract NRF2-mediated cytoprotective response through the promoter binding competition for antioxidant response element (ARE) [33]. However, never has a suppressive transcription factor of NRF2 been discovered. Here, we show for the first time that SOX17 functions as a NRF2 upstream transcriptional suppressor, and dysregulation of SOX17/NRF2 pathway confers CCRT resistance and aggressiveness in ESCC cells. Furthermore, our ChIP analysis identified the recruitment of HDAC1 to *NFE2L2* promoter after SOX17 overexpression, suggesting that HDAC1 may function as a corepressor of SOX17 to transcriptionally suppress NRF2 expression.

Previously, we have demonstrated that SOX17 overexpression could sensitize the resistant ESCC cells to CCRT treatment through transcriptional suppression of DNA repair and damage response genes [24]. Further, the present work revealed that SOX17 also modulates NRF2-mediated cytoprotective programs in ESCC cells. Notably, after SOX17 overexpression, the expressions of NRF2-regulated genes were reduced more significantly in CCRT resistant cells than in the parental cells. Similarly, after ZVI@CMC-induced re-expression of SOX17, these NRF2-targeted genes in CCRT resistant cells were also more responsive to the ZVI@CMC treatment than those in the parental cells. Accordingly, these results suggested that silencing of SOX17 in ESCC may play an important role in the development of NRF2-addiction in the resistant cells, and these resistant cells rely more on the dysregulation of SOX17/NRF2 axis to maintain their resistance, making it a promising therapeutic target for overcoming resistance.

Of note, it has been reported that 4.5% of ESCC patients contain somatic changes in *NFE2L2* [34], and some of the somatic mutations in *NFE2L2* could cause expression alteration [28, 35]. Therefore, *NFE2L2* gene alterations could be partly involved in CCRT resistance in ESCC. Although the genomic analysis of *SOX17* in ESCC has not been reported, the correlation between mutations in *SOX17* gene and CCRT responses is worthy of further investigation. Importantly, our Cox regression analysis showed that SOX17^low^/NRF2^high^ expression pattern could be a valuable independent biomarker for cancer-related death risk of ESCC patients. Indeed, the results of GSEA also identified several inactivation genes, such as the tumor suppressor *TGFBR2* and two pro-ferroptosis genes, *FTH1* and *HMOX1*. We thus suggest that the GSEA-identified inactivated genes are worth further study for the optimization of biomarker establishment. In addition to the development of SOX17/NRF2 as a biomarker, there are databases, such as ClinVar and COSMIC, providing detailed information about genomic variations and their relationship to human health. This information could be helpful for predicting the potential side effects for further development of NRF2 inhibitor/SOX17 agonist.

Indeed, ZVI@CMC could not only exert cancer-specific cytotoxicity due to enhanced lysosomal function in cancer cells [36], but also enhance anticancer immunity to modulate tumor microenvironment [29]. Here, ZVI@CMC was recognized as a DNMT inhibitor to re-express the silenced SOX17 in ESCC, and hence suppressing the activation of NRF2-mediated cytoprotective programs. Consistently, both of our studies showed that ZVI@CMC could trigger AMPK/mTOR signaling to activate GSK3β and promote the nuclear translocation of β-TrCP. Of note, iron overload was recently demonstrated to attenuate DNMT activities and perturb redox-methylation status [37]. Particularly, we previously reported that β-TrCP E3-ligase mediates the ubiquitination and degradation of DNMT1 [38]. Taken together, our findings elucidated a molecular mechanism whereby ZVI@CMC promotes GSK3β/β-TrCP-mediated degradation of DNMT1, and thereby restoring the expression of SOX17 to suppress NRF2 defense pathway. Notably, we previously showed that ZVI@CMC could augment β-TrCP-dependent degradation of NRF2 protein [29]. Therefore, the enhanced nuclear translocation of β-TrCP induced by ZVI@CMC could reduce NRF2 expression both by direct protein degradation and by indirect suppression through SOX17 transcriptional regulation.

At present, azacitidine and decitabine are the only two FDA-approved DNMT inhibitors for anticancer therapy [39]. Unfortunately, as they belong to the family of nucleoside analogs, their poor chemical stability in physiological conditions restricts the efficacy of treatment for solid tumors, and the clinical use is mostly limited to hematological malignancies [40, 41]. Although several other DNMT inhibitors have been developed, the main challenges of these compounds are low cellular activity, weak nuclear penetration, and their ability to integrate everywhere in the genome globally, leading to chromosomal instability and substantial cytotoxicity [42]. Hence, there is a real need to develop novel and more selective DNMT inhibitors. We found that ZVI@CMC could elicit cancer-specific cytotoxicity while sparing non-malignant cells, which could be attributed to the enhanced lysosomal function of cancer cells [29]. In addition, in line with our previous results of animal studies, we demonstrated that ZVI@CMC significantly inhibited tumor growth without showing apparent pathological effects. Genome-wide DNA methylation profiling would be valuable for further characterization of ZVI@CMC for therapeutic applications.

Notably, many ESCC patients harbor an immunologically cold tumor microenvironment [43]. Growing evidence has indicated that DNMT inhibition can promote interferon response and antigen presentation of cancer cells [44]. In addition, DNMT inhibition was discovered to rejuvenate tumor-infiltrating CD8 T cells and suppress the development of regulatory T cells [45–47], which may partly account for our previous observation that ZVI@CMC improves anti-tumor immunity and modulates an immunostimulatory tumor microenvironment [29]. Interestingly, preclinical evidence has shown that radiotherapy can induce viral mimicry state in the irradiated cancer cells due to the accumulation of cytosolic DNA, and thus promoting the recruitment and activation of CD8 T cells [48–50]. Therefore, in addition to overcoming the resistance in ESCC cells, the combination of ZVI@CMC and radiation treatment was suggested to potentiate powerful anti-tumor T-cell responses. The synergistic effects of this combination treatment warrant further investigation.

## Conclusion

We identified a novel transcription regulation of NRF2 by SOX17, and silencing of SOX17 leads to the activation of NRF2-mediating cytoprotective programs, resulting in CCRT resistance of ESCC cells. In addition, ZVI@CMC could serve as a DNMT inhibitor to restore SOX17/NRF2 axis, and the combination of ZVI@CMC with radiation treatment significantly augmented anticancer efficacy to inhibit the tumor growth of CCRT resistant cancer. These results suggested that SOX17/NRF2 axis constitutes a promising therapeutic target for overcoming CCRT resistance in ESCC cells.

## Supplementary Information

**Additional file 1. Supplementary figures and tables.**

## Data Availability

The authors declare that all data supporting the findings of this study are available within the paper in the main text or the supplementary materials. The accession number for RNA-seq dataset is GSE199630.

## Abbreviations

AKR: Aldo–keto reductase
ARE: Antioxidant response element
CCRT: Concomitant chemo-radiation therapy
ChIP: Chromatin immunoprecipitation
DNMT: DNA methyltransferases
ESCC: Esophageal squamous cell carcinoma
GPX2: Glutathione peroxidase 2
GSEA: Gene set enrichment analysis
HDAC1: Histone deacetylase 1
HMG: High mobility group
IHC: Immunohistochemistry
MSP: Methylation-specific PCR
NQO1: NAD(P)H quinone oxidoreductase 1
NRF2: Nuclear factor-erythroid 2-related factor 2
OS: Overall survival
RNA-seq: RNA sequencing
ROS: Reactive oxygen species
tBHQ: Tert-butylhydroquinone
ZVI@CMC: Carboxymethyl cellulose-coated zero-valent-iron

## References

[1] Sung H, Ferlay J, Siegel RL, Laversanne M, Soerjomataram I, Jemal A (2021). Global cancer statistics 2020: GLOBOCAN estimates of incidence and mortality worldwide for 36 cancers in 185 countries. CA Cancer J Clin.

[2] Hirata H, Niida A, Kakiuchi N, Uchi R, Sugimachi K, Masuda T (2021). The evolving genomic landscape of esophageal squamous cell carcinoma under chemoradiotherapy. Cancer Res.

[3] Arnold M, Ferlay J, van Berge Henegouwen MI, Soerjomataram I (2020). Global burden of oesophageal and gastric cancer by histology and subsite in 2018. Gut.

[4] Siegel RL, Miller KD, Fuchs HE, Jemal A (2022). Cancer statistics, 2022. Cancer J Clin.

[5] Chen GZ, Zhu HC, Dai WS, Zeng XN, Luo JH, Sun XC (2017). The mechanisms of radioresistance in esophageal squamous cell carcinoma and current strategies in radiosensitivity. J Thorac Dis.

[6] Stahl M, Stuschke M, Lehmann N, Meyer HJ, Walz MK, Seeber S (2005). Chemoradiation with and without surgery in patients with locally advanced squamous cell carcinoma of the esophagus. J Clin Oncol.

[7] Wang XJ, Hayes JD, Wolf CR (2006). Generation of a stable antioxidant response element-driven reporter gene cell line and its use to show redox-dependent activation of nrf2 by cancer chemotherapeutic agents. Cancer Res.

[8] Chorley BN, Campbell MR, Wang X, Karaca M, Sambandan D, Bangura F (2012). Identification of novel NRF2-regulated genes by ChIP-Seq: influence on retinoid X receptor alpha. Nucleic Acids Res.

[9] Singh A, Rangasamy T, Thimmulappa RK, Lee H, Osburn WO, Brigelius-Flohe R (2006). Glutathione peroxidase 2, the major cigarette smoke-inducible isoform of GPX in lungs, is regulated by Nrf2. Am J Respir Cell Mol Biol.

[10] He F, Ru X, Wen T (2020). NRF2, a transcription tactor for stress response and beyond. Int J Mol Sci.

[11] Tu W, Wang H, Li S, Liu Q, Sha H (2019). The anti-inflammatory and anti-oxidant mechanisms of the Keap1/Nrf2/ARE signaling pathway in chronic diseases. Aging Dis.

[12] Cloer EW, Goldfarb D, Schrank TP, Weissman BE, Major MB (2019). NRF2 activation in cancer: from DNA to protein. Cancer Res.

[13] Hayashi M, Kuga A, Suzuki M, Panda H, Kitamura H, Motohashi H (2020). Microenvironmental activation of Nrf2 restricts the progression of Nrf2-activated malignant tumors. Cancer Res.

[14] Mitsuishi Y, Taguchi K, Kawatani Y, Shibata T, Nukiwa T, Aburatani H (2012). Nrf2 redirects glucose and glutamine into anabolic pathways in metabolic reprogramming. Cancer Cell.

[15] Owusu-Ansah E, Banerjee U (2009). Reactive oxygen species prime Drosophila haematopoietic progenitors for differentiation. Nature.

[16] Niture SK, Jaiswal AK (2012). Nrf2 protein up-regulates antiapoptotic protein Bcl-2 and prevents cellular apoptosis. J Biol Chem.

[17] Dodson M, Castro-Portuguez R, Zhang DD (2019). NRF2 plays a critical role in mitigating lipid peroxidation and ferroptosis. Redox Biol.

[18] Itoh K, Wakabayashi N, Katoh Y, Ishii T, Igarashi K, Engel JD (1999). Keap1 represses nuclear activation of antioxidant responsive elements by Nrf2 through binding to the amino-terminal Neh2 domain. Genes Dev.

[19] Rada P, Rojo AI, Chowdhry S, McMahon M, Hayes JD, Cuadrado A (2011). SCF/{beta}-TrCP promotes glycogen synthase kinase 3-dependent degradation of the Nrf2 transcription factor in a Keap1-independent manner. Mol Cell Biol.

[20] Zhang J, Jiao Q, Kong L, Yu J, Fang A, Li M (2018). Nrf2 and Keap1 abnormalities in esophageal squamous cell carcinoma and association with the effect of chemoradiotherapy. Thorac Cancer.

[21] Li AF, Hsu PK, Tzao C, Wang YC, Hung IC, Huang MH (2009). Reduced axin protein expression is associated with a poor prognosis in patients with squamous cell carcinoma of esophagus. Ann Surg Oncol.

[22] DeNicola GM, Karreth FA, Humpton TJ, Gopinathan A, Wei C, Frese K (2011). Oncogene-induced Nrf2 transcription promotes ROS detoxification and tumorigenesis. Nature.

[23] Kuo IY, Wu CC, Chang JM, Huang YL, Lin CH, Yan JJ (2014). Low SOX17 expression is a prognostic factor and drives transcriptional dysregulation and esophageal cancer progression. Int J Cancer.

[24] Kuo IY, Huang YL, Lin CY, Lin CH, Chang WL, Lai WW (2019). SOX17 overexpression sensitizes chemoradiation response in esophageal cancer by transcriptional down-regulation of DNA repair and damage response genes. J Biomed Sci.

[25] Chang WL, Wang WL, Chung TJ, Lin FC, Yen CJ, Lai WW (2015). Response evaluation with endoscopic ultrasound and computed tomography in esophageal squamous cell carcinoma treated by definitive chemoradiotherapy. J Gastroenterol Hepatol.

[26] Wu SM, Tsai WS, Chiang SF, Lai YH, Ma CP, Wang JH (2020). Comprehensive transcriptome profiling of Taiwanese colorectal cancer implicates an ethnic basis for pathogenesis. Sci Rep.

[27] Martens M, Ammar A, Riutta A, Waagmeester A, Slenter DN, Hanspers K (2021). WikiPathways: connecting communities. Nucleic Acids Res.

[28] Jiang X, Zhou X, Yu X, Chen X, Hu X, Lu J (2022). High expression of nuclear NRF2 combined with NFE2L2 alterations predicts poor prognosis in esophageal squamous cell carcinoma patients. Mod Pathol.

[29] Hsieh CH, Hsieh HC, Shih FS, Wang PW, Yang LX, Shieh DB (2021). An innovative NRF2 nano-modulator induces lung cancer ferroptosis and elicits an immunostimulatory tumor microenvironment. Theranostics.

[30] Kitamura H, Motohashi H (2018). NRF2 addiction in cancer cells. Cancer Sci.

[31] Yang L, Shen C, Estrada-Bernal A, Robb R, Chatterjee M, Sebastian N (2021). Oncogenic KRAS drives radioresistance through upregulation of NRF2-53BP1-mediated non-homologous end-joining repair. Nucleic Acids Res.

[32] Tang YC, Hsiao JR, Jiang SS, Chang JY, Chu PY, Liu KJ (2021). c-MYC-directed NRF2 drives malignant progression of head and neck cancer via glucose-6-phosphate dehydrogenase and transketolase activation. Theranostics.

[33] Faraonio R, Vergara P, Di Marzo D, Pierantoni MG, Napolitano M, Russo T (2006). p53 suppresses the Nrf2-dependent transcription of antioxidant response genes. J Biol Chem.

[34] Song Y, Li L, Ou Y, Gao Z, Li E, Li X (2014). Identification of genomic alterations in oesophageal squamous cell cancer. Nature.

[35] Huppke P, Weissbach S, Church JA, Schnur R, Krusen M, Dreha-Kulaczewski S (2017). Activating de novo mutations in NFE2L2 encoding NRF2 cause a multisystem disorder. Nat Commun.

[36] Yang LX, Wu YN, Wang PW, Huang KJ, Su WC, Shieh DB (2020). Silver-coated zero-valent iron nanoparticles enhance cancer therapy in mice through lysosome-dependent dual programed cell death pathways: triggering simultaneous apoptosis and autophagy only in cancerous cells. J Mater Chem B.

[37] Ye Q, Trivedi M, Zhang Y, Bohlke M, Alsulimani H, Chang J (2019). Brain iron loading impairs DNA methylation and alters GABAergic function in mice. FASEB J.

[38] Lin RK, Hsieh YS, Lin P, Hsu HS, Chen CY, Tang YA (2010). The tobacco-specific carcinogen NNK induces DNA methyltransferase 1 accumulation and tumor suppressor gene hypermethylation in mice and lung cancer patients. J Clin Invest.

[39] Ganesan A, Arimondo PB, Rots MG, Jeronimo C, Berdasco M (2019). The timeline of epigenetic drug discovery: from reality to dreams. Clin Epigenetics.

[40] Wee S, Dhanak D, Li H, Armstrong SA, Copeland RA, Sims R (2014). Targeting epigenetic regulators for cancer therapy. Ann N Y Acad Sci.

[41] Juo YY, Gong XJ, Mishra A, Cui X, Baylin SB, Azad NS (2015). Epigenetic therapy for solid tumors: from bench science to clinical trials. Epigenomics.

[42] Erdmann A, Halby L, Fahy J, Arimondo PB (2015). Targeting DNA methylation with small molecules: what's next?. J Med Chem.

[43] van Duijvenvoorde M, Derks S, Bahce I, Leemans CR, van de Ven R, Fransen MF (2022). Comparison of the tumor microenvironments of squamous cell carcinoma at different anatomical locations within the upper aerodigestive tract in relation to response to ICI therapy. Clin Transl Immunol.

[44] Chiappinelli KB, Zahnow CA, Ahuja N, Baylin SB (2016). Combining epigenetic and immunotherapy to combat cancer. Cancer Res.

[45] Travers M, Brown SM, Dunworth M, Holbert CE, Wiehagen KR, Bachman KE (2019). DFMO and 5-azacytidine increase M1 macrophages in the tumor microenvironment of murine ovarian cancer. Cancer Res.

[46] Wang L, Amoozgar Z, Huang J, Saleh MH, Xing D, Orsulic S (2015). Decitabine enhances lymphocyte migration and function and synergizes with CTLA-4 blockade in a murine ovarian cancer model. Cancer Immunol Res.

[47] Costantini B, Kordasti SY, Kulasekararaj AG, Jiang J, Seidl T, Abellan PP (2013). The effects of 5-azacytidine on the function and number of regulatory T cells and T-effectors in myelodysplastic syndrome. Haematologica.

[48] Lhuillier C, Rudqvist NP, Elemento O, Formenti SC, Demaria S (2019). Radiation therapy and anti-tumor immunity: exposing immunogenic mutations to the immune system. Genome Med.

[49] Deng L, Liang H, Xu M, Yang X, Burnette B, Arina A (2014). STING-dependent cytosolic DNA sensing promotes radiation-induced type I interferon-dependent antitumor immunity in immunogenic tumors. Immunity.

[50] Burnette BC, Liang H, Lee Y, Chlewicki L, Khodarev NN, Weichselbaum RR (2011). The efficacy of radiotherapy relies upon induction of type i interferon-dependent innate and adaptive immunity. Cancer Res.

