# Supplementary material for: Dysregulation of SOX17/NRF2 axis confers chemoradiotherapy resistance and emerges as a novel therapeutic target in esophageal squamous cell carcinoma
Source: J Biomed Sci. 2022 Oct 30;29:90. doi: 10.1186/s12929-022-00873-4 (PMC9618214; doi:10.1186/s12929-022-00873-4)
Supplement: Supplementary file 1 — Additional file 1. Supplementary figures and tables. [file 12929_2022_873_MOESM1_ESM.pdf]

Additional file 1: Supplementary Information

Dysregulation of SOX17/NRF2 axis confers chemoradiotherapy resistance and emerges as a novel therapeutic target in esophageal squamous cell carcinoma

Chih-Hsiung Hsieh, Wen-Hui Kuan, Wei-Lun Chang, I-Ying Kuo, Hsun Liu, Dar-Bin Shieh, Hsuan Liu, Bertrand Tan, and Yi-Ching Wang

Supplementary Figures and Tables

Supplementary Figure S1 is related to Figure 1.

Supplementary Figure S2 is related to Figure 2.

Supplementary Figure S3 is related to Figure 3.

Supplementary Figure S4 is related to Figure 4.

Supplementary Figure S5 is related to Figure 5.

Supplementary Figure S6 is related to Figure 6.

Supplementary Table S1 is related to Figure 1 and Materials and Methods.

Supplementary Table S2 is related to Figure 1 and Materials and Methods.

Supplementary Table S3 is related to Materials and Methods.

Supplementary Table S4 is related to Materials and Methods.

Supplementary Table S5 is related to Materials and Methods.

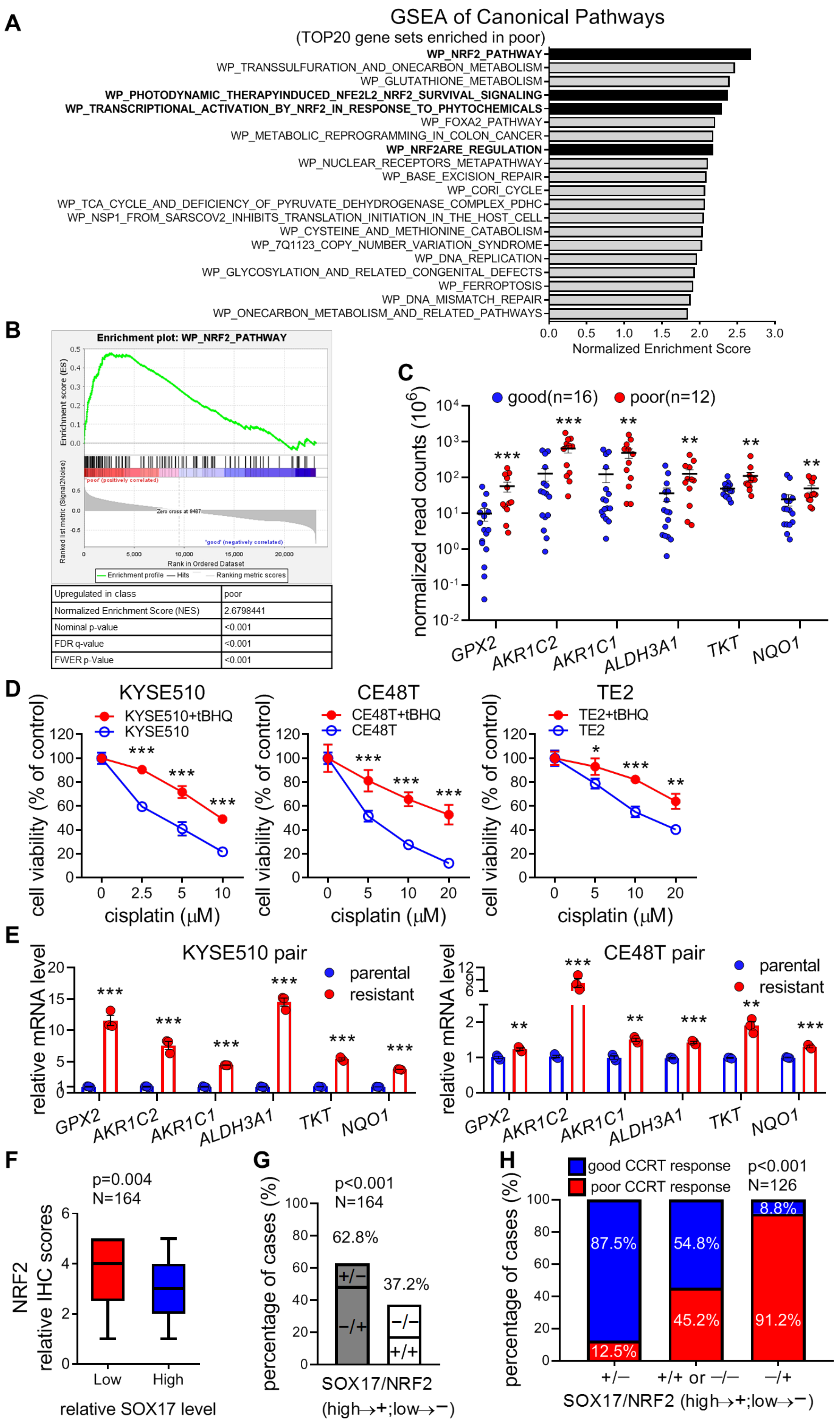

Supplementary Figure S1, related to Figure 1.

Expression signature analysis of ESCC cells and patients. **A,** Gene set enrichment analysis (GSEA) of RNA-seq data by using WikiPathways pathway database. **B,** The GSEA-revealed enrichment plot of NRF2 pathway. **C,** RNA-seq read count data of NRF2 downstream genes (good, n=16; poor, n=12). **D,** MTT assay of tBHQ-treated ESCC cells incubated with various concentrations of cisplatin for 72 h. **E,** Basal mRNA expression levels of NRF2 downstream genes in the resistant and parental cells were examined by RT-qPCR analysis. *β-actin* was used as an internal control. **F,** Qualitative scoring of immunohistochemistry staining revealed an inverse correlation between SOX17 and NRF2 protein expression (n=164). The qualitative scores are defined in the Methods and materials section. **G,** Most ESCC patients showed an inverse expression of SOX17 and NRF2 (gray bar) (n=164). **H,** $SOX17^{low}/NRF2^{high}$ is significantly associated with poor CCRT response (n=126). Data represents mean ± s.e.m. ns: non-significant; *, p<0.05; **, p<0.01; ***, p<0.001.

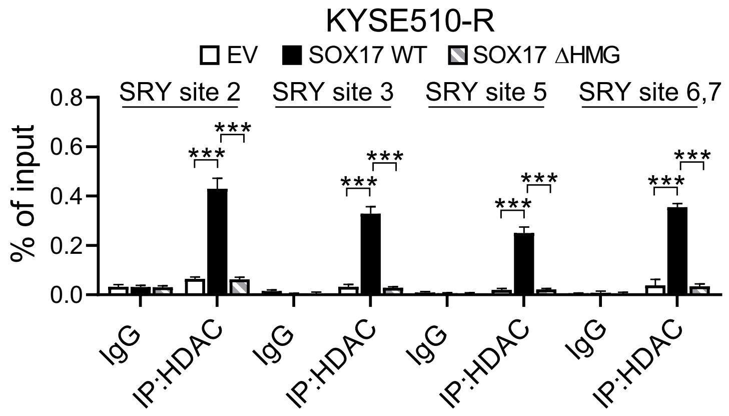

Supplementary Figure S2, related to Figure 2.

ChIP-qPCR assay was performed to measure HDAC binding ability to the promoter region of *NFE2L2* in KYSE510-R cells. Data represents mean ± s.e.m. ns: non-significant; *, $p<0.05$; **, $p<0.01$; ***, $p<0.001$.

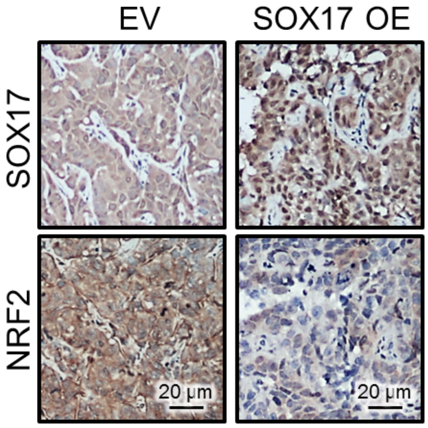

Supplementary Figure S3, related to Figure 3.

Immunohistochemistry staining revealed a decreased expression of NRF2 protein in the SOX17-overexpressing tumor xenografts.

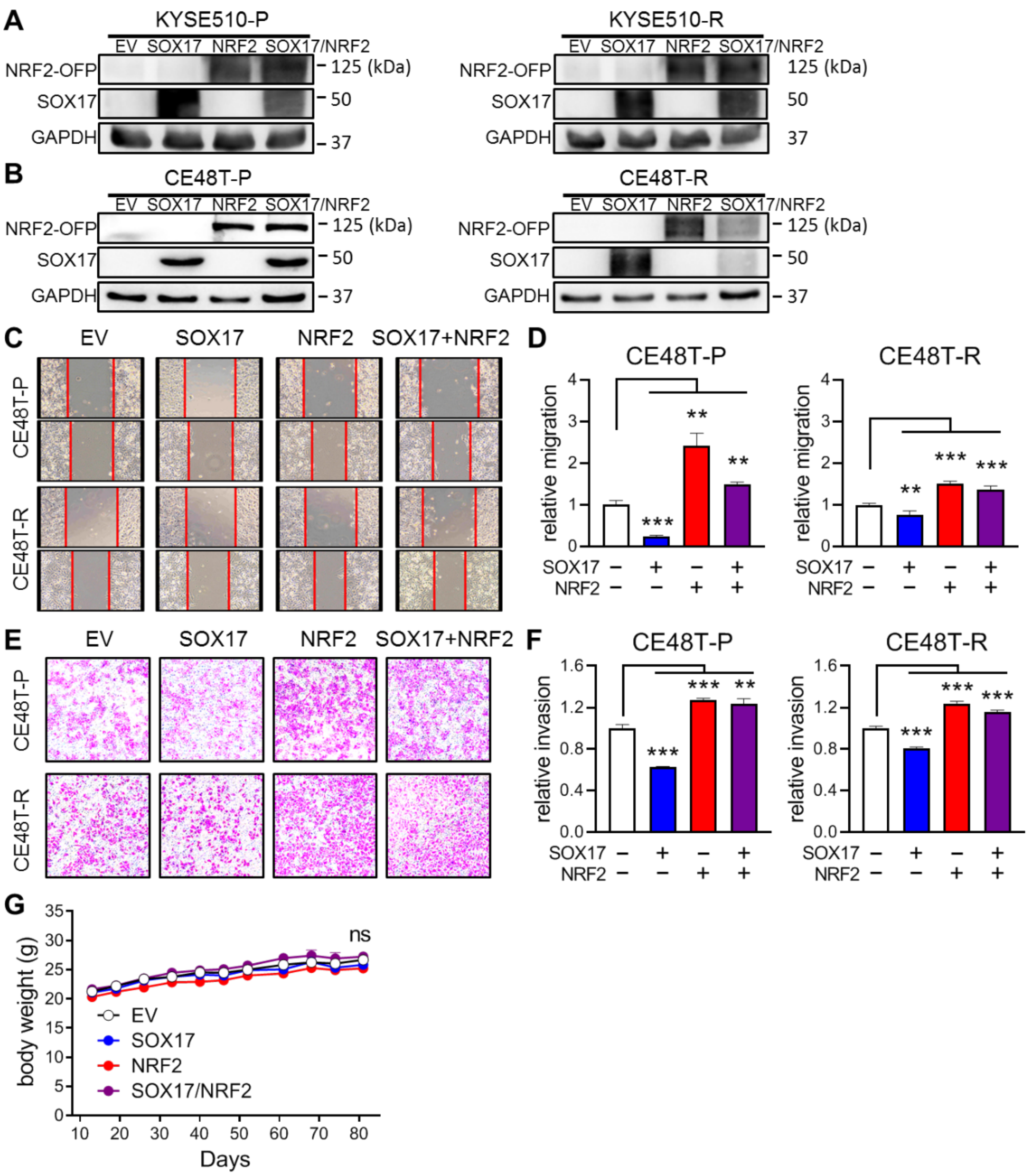

Supplementary Figure S4, related to Figure 4.

The effects of SOX17 and/or NRF2 overexpression on cell behaviors and tumor growth. **A** and **B,** Western blots analysis confirmed the ectopic overexpression of SOX17 and NRF2 in KYSE510 pair (**A**) and CE48T pair (**B**) cells. GAPDH was used as an internal control. **C** and **D,** Wound healing assay of CE48T pair cells manipulated with SOX17 and/or NRF2 expression. Cells were monitored for their ability to migrate into the wound gap. The wound gap was photographed (**C**) and quantified (**D**) at 24 h. **E** and **F,** Transwell invasion assay of CE48T pair cells manipulated with SOX17 and/or NRF2 expression. The invaded cells were photographed (**E**) and quantified (**F**) at 20 h (n=3). **G,** Body weight was recorded during the animal experiment. Data represents mean ± s.e.m. ns: non-significant; *, p<0.05; **, p<0.01; ***, p<0.001.

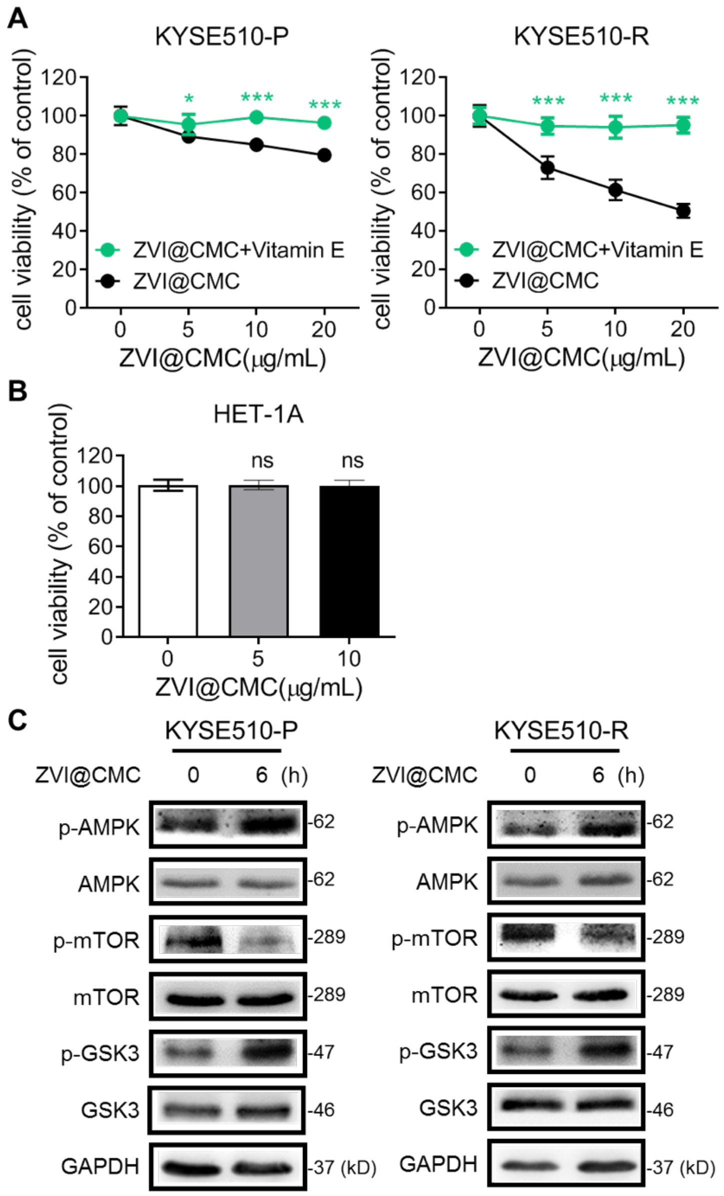

Supplementary Figure S5, related to Figure 5.

The effects of ZVI@CMC treatment on ESCC and non-tumorigenic esophageal epithelial cells. **A,** Cell viability was determined by MTT assay after co-treatment with ZVI@CMC and Vitamin E (100 μM) for 48 h. **B,** MTT assay of non-tumorigenic esophageal epithelial cells HET-1A treated with various concentrations of ZVI@CMC for 72 h. **C,** Immunoblotting of p-AMPK, total AMPK, p-mTOR, total mTOR, p-GSK3β and total GSK3β in cells treated with ZVI@CMC for the indicated time. GAPDH was used as an internal control. Data represents mean ± s.e.m. ns: non-significant; *, $p<0.05$; **, $p<0.01$; ***, $p<0.001$.

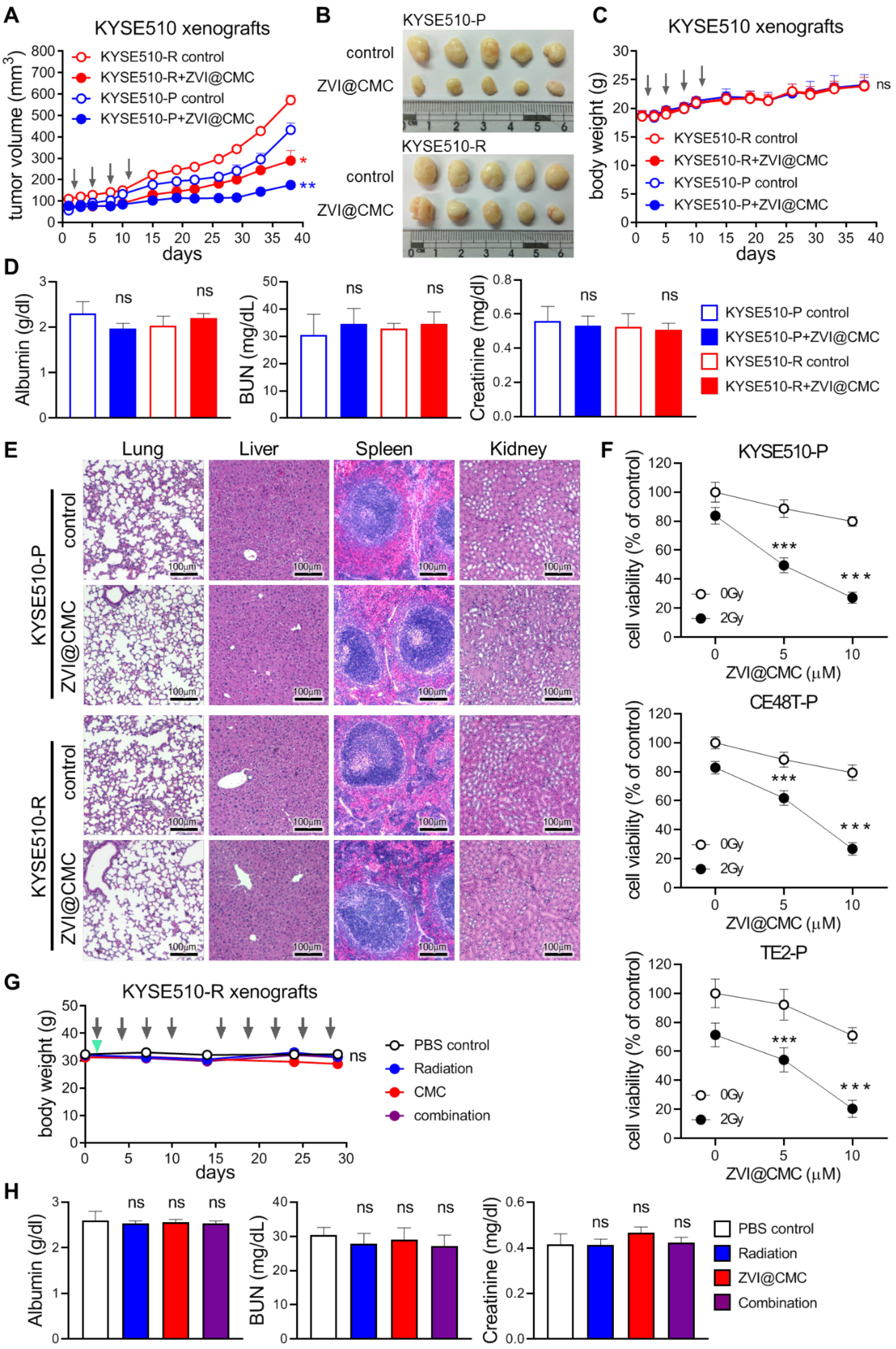

Supplementary Figure S6, related to Figure 6.

Animal studies on ESCC tumor xenografts after treatment with ZVI@CMC alone or in combination with radiation. **A,** Tumor growth of KYSE510-P and -R xenografts treated with ZVI@CMC. **B** and **C,** Tumor weight (**B**) and tumor size (**C**) were measured at the end of the experiment. **D** and **E,** The blood biochemistry analysis (**D**) and the H&E staining of major organs (**E)** of the mice with KYSE510-P or -R xenografts after ZVI@CMC treatment. **F,** MTT assay of ESCC parental cells treated with the combination of ZVI@CMC and radiation treatment for 72 h. **G,** Body weight of the mice treated with the combination treatment was recorded during the animal experiment. **H,** The blood biochemistry analysis of the mice treated with the combination treatment. Data represents mean ± s.e.m. ns: non-significant; *, $p<0.05$; **, $p<0.01$; ***, $p<0.001$.

Supplementary Table S1. The information of patients whose tumor tissues used for RNA-seq.

Patient number[a]	SCC antigen marker	Radiotherapy dose	Chemotherapy regimen
G1	1.10	5940	cisplatin+5-FU+Leucovorin
G2	1.44	6660	cisplatin+5-FU
G3	7.03	3600	cisplatin+5-FU
G4	0.91	6660	cisplatin+5-FU
G5	4.17	5040	cisplatin+5-FU
G6	3.33	5000	cisplatin+5-FU
G7	-[b]	5000	cisplatin
G8	0.90	6300	cisplatin+5-FU
G9	1.00	5000	cisplatin+5-FU
G10	1.27	6660	cisplatin+5-FU
G11	1.50	5040	cisplatin+5-FU
G12	1.05	6660	ciaplatin+5-FU
G13	0.40	3600	cisplatin+5-FU
G14	2.48	5000	cisplatin+5-FU
G15	1.36	6120	cisplatin+5-FU
G16	-[b]	5600	cisplatin+5-FU
P1	1.15	3600	cisplatin+Taxol
P2	1.30	3600	cisplatin+5-FU+Leucovorin
P3	4.62	5040	cisplatin+5-FU
P4	-[b]	6660	cisplatin+5-FU
P5	1.64	5400	cisplatin+5-FU+Leucovorin
P6	-[b]	5000	cisplatin+5-FU+LV
P7	1.86	3600	cisplatin+5-FU
P8	5.20	6600	cisplatin+5-FU
P9	1.39	3780	cisplatin+5-FU
P10	1.01	5040	cisplatin+5-FU
P11	-[b]	3780	carboplatin+5-FU+LV
P12	-[b]	4140	cisplatin+5-FU

[a] G: good responder; P: poor responder.

[b] The SCC antigen marker is not applicable to this patient.

Supplementary Table S2. The raw FPKM revealed by TCGA-ESCA dataset

	SOX17	NFE2L2	GPX2	AKR1C1	AKR1C2	TKT	ALDH3A	NQO1
TCGA-Z6-AAPN	0.32838783	40.5866298	2.8361088	0.13784433	0.17848029	21.6083898	2.8708162	7.55336581
TCGA-VR-A8EZ	1.49178382	64.1014541	992.941689	218.444233	156.270883	108.536211	188.139443	96.5424821
TCGA-JY-A93F	0.24436005	36.9371441	4.22823572	1.48838476	12.9262855	27.0900767	1.51909871	12.9618511
TCGA-LN-A9FR	0.42945159	43.761479	324.584324	54.3317543	30.9858214	74.2679162	14.9078857	78.846979
TCGA-LN-A8I0	0.18109721	39.9587763	1276.43591	132.688008	169.462808	83.4278082	1023.80729	166.165045
TCGA-IG-A3YA	1.89383956	71.8172224	193.495492	10.9822981	53.3174335	24.0240964	24.6967646	19.339627
TCGA-LN-A9FO	0.96768884	53.5838782	10.4807482	2.65242462	7.95409652	39.5032413	2.17160038	21.0004698
TCGA-VR-AA4G	1.13321165	73.3973778	29.5425701	0.80984961	3.03553526	17.6221938	20.7304993	17.8133062
TCGA-IG-A3I8	0.18922865	35.2382052	31.1413892	13.5578991	23.8084313	18.5912153	10.2061986	15.1349342
TCGA-V5-A7RC	0.74343899	117.692785	347.196934	152.32	122.593713	48.2775389	165.296643	131.835351
TCGA-L5-A43J	0.4788249	53.1508417	77.2058462	1.96568167	12.6556087	37.8483947	1.33279981	46.5939204
TCGA-LN-A4A4	0.61680001	63.4787069	284.720792	15.7900946	31.3680877	27.8724181	337.374191	76.7960214
TCGA-Z6-A8JE	0.72130589	20.7172562	51.1599628	3.772937	90.5216302	15.6959459	2.59724649	19.303136
TCGA-Z6-A8JD	1.57347327	24.5900034	3.97675102	1.24898645	1.26122546	20.2472857	3.64878025	11.2048432
TCGA-LN-A49O	0.32755933	22.4711156	62.7694241	2.06310621	0.30866238	26.9244096	1.03536605	9.75164813
TCGA-L5-A4OM	0.23891406	59.80412	54.9956039	8.78300793	28.7953604	17.7167337	44.8524331	15.1892267
TCGA-LN-A9FP	6.91498527	37.4745631	78.2840831	9.5019741	8.30904799	17.2905535	10.2053697	10.659379
TCGA-LN-A4A9	0.48217127	32.4374705	2.84467272	1.73046696	0.97309483	13.3973637	0.67538069	6.61952346
TCGA-IG-A3YB	0.78770383	117.174274	280.899963	4.23924474	170.689577	23.1826149	8.55503827	51.2333654
TCGA-L5-A8NK	0.78102764	59.4578613	27.4906693	3.66939636	5.45775541	21.8637496	69.7192239	24.3562327
TCGA-VR-A8EX	0.24773767	64.4708893	158.694237	4.10899518	15.7401219	25.3880163	89.4209081	23.9484724
TCGA-LN-A4A1	0.35895793	36.3538316	24.5006005	11.1114715	16.1621251	16.5623236	24.5280644	16.692665
TCGA-IG-A97I	0.25810954	62.902372	262.722749	0.62207231	1.72330346	23.2728793	4.19377401	36.964667
TCGA-LN-A7HX	0.4760527	37.0523611	451.557126	78.8889705	223.578691	55.3104884	34.6015751	64.7350493
TCGA-LN-A49M	0.56442504	24.1257439	52.2167022	10.6989661	16.2001306	20.2877667	2.47786085	21.3072743
TCGA-IG-A51D	1.0270049	38.4816812	37.4451851	0.72650634	0.1794156	26.2791567	2.6014613	24.608366
TCGA-V5-A7RC	0.77610862	131.219649	277.608271	90.2586393	98.0663637	32.2552972	335.608405	91.358208
TCGA-LN-A7HV	1.06501109	22.7719547	28.8550262	4.03401578	7.17259495	38.9470977	3.51853454	20.319664
TCGA-LN-A49Y	1.20319398	15.1058051	52.3080667	1.14627453	6.75880104	18.2468021	11.0573347	5.05617241
TCGA-LN-A4A3	0.63564581	29.9637337	23.5840226	4.73210408	6.42720709	26.6484373	7.28831979	19.9263654
TCGA-KH-A6WC	0.54379058	81.6634721	50.6930844	7.92130677	7.64785313	24.8924196	173.250306	34.7237072
TCGA-LN-A4A5	1.63115346	33.1198717	9.1600784	1.09333499	4.14355591	12.9140361	1.26631051	8.94520831
TCGA-Z6-A9VB	0.31332373	195.350856	418.796624	279.182984	314.79147	75.6180999	648.838986	72.4250818
TCGA-JY-A6FA	0.51433169	168.357756	615.534161	283.889752	197.157985	168.919205	366.020235	144.921997
TCGA-IG-A97H	0.15678335	118.840964	48.8047081	7.59644807	16.4382148	28.4850593	53.3442089	23.9720714
TCGA-VR-A8EU	0.39896887	44.1440198	112.174298	77.5758128	53.1484921	38.9541244	2.21450564	75.8321319
TCGA-LN-A49P	0.75428962	110.780049	166.418661	53.5108862	82.8323883	51.9221107	243.880778	72.7310172
TCGA-LN-A5U6	0.51225719	119.844623	364.729933	102.012792	203.901752	52.864308	19.236945	95.7670925
TCGA-VR-A8ER	0.75174286	92.3378208	248.790628	160.866617	82.4259562	51.1875582	51.1407649	125.730974
TCGA-IG-A625	0.47030305	94.3609876	497.711007	307.154549	279.654217	114.458791	434.508822	210.801785

TCGA-LN-A49X	1.08132275	29.0275277	69.8371508	1.26830514	0.48575523	22.4230511	3.88098784	11.6098866
TCGA-LN-A49W	1.15263035	31.5911041	14.8283863	0.70058937	0.63060716	22.0742604	1.70734923	15.7742009
TCGA-LN-A49U	0.37046541	59.1295817	287.447505	126.382603	120.831358	46.2452832	127.903692	77.8682021
TCGA-IC-A6RF	0.54063294	114.30814	345.60722	80.3313062	92.4668196	63.6728016	1052.19829	66.3603384
TCGA-IG-A8O2	0.8354442	68.218745	687.081527	434.118759	329.533984	186.916792	1889.33934	244.734128
TCGA-S8-A6BW	0.23126681	131.04069	389.18624	81.1952007	135.738591	35.192033	167.742688	54.5716777
TCGA-LN-A49S	0.37362929	68.0252629	57.6243691	12.3482576	20.7447083	28.8114218	37.5904612	22.0053872
TCGA-LN-A5U5	0.44231874	28.527059	502.947417	185.17404	140.828965	64.4417215	719.942402	399.728538
TCGA-VR-A8EY	0.31441518	29.4811837	245.635735	97.6042902	117.14725	75.8323837	93.482099	72.5079821
TCGA-L5-A88Z	0.87834925	93.6923985	1592.37591	207.344107	304.196699	61.5867182	1111.78238	138.812675
TCGA-IC-A6RF	0.70444908	40.7529871	30.4528924	5.56459783	3.97277112	42.4751035	9.07413313	42.6283532
TCGA-V5-AASV	0.49983244	37.9241021	9.31025662	0.02581395	0.04082478	54.4879198	0.75664259	2.51820928
TCGA-VR-A8EW	0.26160383	147.583332	424.745221	59.443599	87.8860809	63.8995413	125.378128	108.172931
TCGA-IG-A5S3	1.09651887	34.2388722	14.1628839	0.4905569	0.6378062	11.9068427	9.41462381	8.79775346
TCGA-IG-A3QL	0.46678054	196.103011	305.299227	146.060399	170.417468	84.4086596	260.954744	52.6133063
TCGA-XP-A8T6	1.99402475	128.489301	474.779545	213.931671	137.857328	81.8232533	275.311585	97.5687027
TCGA-LN-A7HY	1.24666749	17.9425342	1.37105036	0.64993476	0.89549485	17.1020838	2.5894674	11.3035144
TCGA-JY-A6FD	2.37078765	29.3115386	18.4591439	1.56241683	1.29330948	21.7496527	6.74482089	5.86750768
TCGA-IG-A50L	0.36054385	25.9150764	342.348408	102.207735	86.6725814	63.9601352	146.886475	128.867636
TCGA-IG-A4P3	0.69300927	74.3271081	394.509498	14.3780746	53.8895433	21.9918746	85.7959747	27.4715578
TCGA-IG-A6QS	0.40152614	38.4901724	188.873105	11.7572619	4.15791607	27.032159	14.5190981	35.7803416
TCGA-LN-A7HZ	0.854233	61.0271215	1544.88813	230.676288	70.5273782	186.436457	478.200642	175.151833
TCGA-IG-A5B8	0.08650992	20.1199949	5.9893186	3.0058953	5.68063679	18.4089402	0.46540482	19.9516392
TCGA-LN-A8I1	0.34591578	27.0550956	282.556154	123.549171	157.544222	43.7446298	930.472142	243.818825
TCGA-VR-AA7I	0.77069794	35.8771642	7.06908037	2.0654523	0.95438204	13.0187952	7.73140589	27.4366038
TCGA-LN-A4MQ	0.472284	56.6695604	452.995807	88.5015844	76.0443192	49.8838974	248.228781	144.460319
TCGA-L5-A88S	4.57143415	42.5967561	2.36354396	4.77983053	6.39473608	13.6807761	9.06252313	15.6953668
TCGA-LN-A5U7	0.25237444	99.8782873	411.206463	159.908946	64.446886	66.6556626	289.689707	143.263612
TCGA-L7-A56G	0.69206314	42.6214173	21.0497313	1.13487726	4.31932055	22.7942739	11.9934847	11.5365994
TCGA-LN-A8HZ	0.30189361	50.7945209	806.170807	195.970544	167.64806	96.1822532	157.258881	134.219572
TCGA-VR-A8ET	0.31170165	46.6593193	62.3201199	1.43450701	17.2345713	15.4723949	1.30133498	11.1609592
TCGA-L5-A8NQ	1.03405497	29.2644127	58.3881266	6.93918012	13.2324094	30.4924633	35.7538261	33.9792043
TCGA-L5-A88W	0.34626084	31.5808074	3.58049462	14.6550558	18.5405037	43.2524868	0.94774496	17.0547744
TCGA-VR-A8EP	0.33372738	39.3333788	285.891622	151.597413	126.664878	30.5373323	19.5220909	30.6782475
TCGA-JY-A6FE	0.37397018	27.1731027	2.06383352	8.59354681	7.41832103	71.7539628	4.17879613	86.8119432
TCGA-LN-A9FQ	0.29519213	77.2773998	529.243799	147.751635	106.947914	117.646879	298.928389	143.537698
TCGA-XP-A8T8	0.56973033	107.267561	271.364593	59.9080065	133.955326	85.3923584	587.585666	115.598939
TCGA-VR-A8EO	2.13475924	24.488175	10.9991523	7.756537	3.99826892	14.8583638	0.28401353	7.33546188
TCGA-LN-A7HW	0.35468665	75.744559	469.315261	317.704793	296.128118	58.3319686	415.741907	228.437761
TCGA-IG-A3YC	0.92795867	44.0804577	25.1570505	4.9278538	6.49250264	19.9613849	37.7600738	12.8587376
TCGA-VR-A8Q7	0.34019384	19.607245	1.66912893	0.21518897	0.01251182	29.6052133	0.11059506	4.14522636
TCGA-LN-A4A8	1.03490526	37.2301398	49.7018341	4.14460447	1.70316997	16.3923485	3.47941464	22.4793266

Supplementary Table S3. The characteristics of plasmids used in the current study.

Plasmid	Target	Insert (bp)	Function	Source
pcDNA3.1/V5-His B	None	-[a]	Vector control	Stephen B. Baylin
pcDNA3.1/V5-His B-SOX17-WT	*SOX17*	1,242	Overexpression	Stephen B. Baylin
pcDNA3.1/V5-His B-SOX17-ΔHMG	SOX17 mut_135-414	837	Overexpression	Stephen B. Baylin
pGL4-vector	None	-[a]	Vector control	Promega
pCMV3-C-OFP/NFE2L2	*NRF2*	2,505	Overexpression	Sino Biological Inc
pGL4-NRF2-Luc	*NRF2* promoter	2,010	Promoter activity	Homemade [b]

[a] The plasmid is used as a backbone vector therefore there is no insert DNA fragment.

[b] The DNA fragment corresponding to residues − 2000 ~ + 10 bp of the transcription start site were PCR amplified with the primers listed in **Table S4**. The PCR product was restricted by *KpnI* and *XhoI* enzymes and then cloned into the pGL4 basic vector to generate pGL4-NRF2-Luc promoter plasmid.

Supplementary Table S4. The primers used in the current study.

Gene	Primer	Sequences (5'→ 3')	Application[a]	PCR size (bp)	Tm (°C)
NFE2L2 promoter pGL4-NRF2-Luc (2010 bp)	Forward	ATG GTA CCT TTT TAT GAG CAA TCT GGA GCA	Promoter construct	2,010	60
	Reverse	ATC TCG AGT CCC TGA TTT GGA GTT GCA G			
NFE2L2 DNA *(SRY 2)*	Forward	CCA GCA CCT CCT CTT TCT TG	ChIP-qPCR	194	60
	Reverse	GAA CCC CAT TCT CAA GAC CA			
NFE2L2 DNA (SRY 3)	Forward	TGG TCT TGA GAA TGG GGT TC	ChIP-qPCR	71	60
	Reverse	TGC TGC AAT TTG CTG AGA GT			
NFE2L2 DNA (SRY 5)	Forward	GCT TTG GTG GGA AGA GGT TC	ChIP-qPCR	83	60
	Reverse	AGC TCG TGT TCG CAG TCA C			
NFE2L2 DNA (SRY 6,7)	Forward	AGG GGT GTG TGT GTG GTT TT	ChIP-qPCR	173	60
	Reverse	TTG CCC AGA GAA GAC TTC AA			
β-actin mRNA	Forward	GGC GGC ACC ACC ATG TAC CCT	RT-qPCR	180	60
	Reverse	AGG GGC CGG ACT CGT CAT ACT			
NFE2L2 mRNA	Forward	CAG CGA CGG AAA GAG TAT GAG C	RT-qPCR	201	60
	Reverse	GTG GGC AAC CTG GGA GTA GTT			
G6PD mRNA	Forward	AGT ACG ATG ATG CAG CCT CCT AC	RT-qPCR	179	60
	Reverse	CTC CAC GAT GAT GCG GTT C			
GPX2 mRNA	Forward	TTG GAC ATC AGG AGA ACT GTC AGA	RT-qPCR	149	60
	Reverse	CTT CAG GTA GGC GAA GAC AGG AT			
NQO1 mRNA	Forward	GGC AGA AGA GCA CTG ATC GTA CT	RT-qPCR	143	60
	Reverse	ATG GGA TTG AAG TTC ATG GCA			
AKR1C1 mRNA	Forward	ATT TGC CAG CCA GGC TAG TG	RT-qPCR	179	60

	Reverse	AGA ATC AAT ATG GCG GAA GCC			
AKR1C2 mRNA	Forward	AAG TAA AGC TCT AGA GGC CGT	RT-qPCR	86	60
	Reverse	GCT CCT CAT TAT TGT AAA CAT GT			
AKR1C3 mRNA	Forward	GGG ATC AAC GAG AGA CAA ACG	RT-qPCR	68	60
	Reverse	AAA GGA CTG GGT CCT CCA AGA			
ALDH3A1 mRNA	Forward	TCC AGC AAC GAC AAG GTG ATT	RT-qPCR	154	60
	Reverse	AGC TCT TCT TGC CAT GGT AGG AT			
TKT mRNA	Forward	GAA GAT CAG CTC CGA CTT GGA	RT-qPCR	128	60
	Reverse	TGT CGA AGT ATT TGC CGG TGT A			
SOX17 DNA (U primer)	Forward	TTA AAT GAT TTT GGG TAA GTA TGT T	MSP	117	53
	Reverse	AAC CAA AAC TAA ACT CTA ACA CCA C			
SOX17 DNA (M primer)	Forward	TTA AAC GAT TTT GGG TAA GTA CGT C	MSP	117	57
	Reverse	AAC CGA AAC TAA ACT CTA ACG CCG			

[a.] RT-qPCR: Reverse transcriptase-quantitative polymerase chain reaction; ChIP-qPCR: Chromatin immunoprecipitation-qPCR; MSP: Methylation specific-PCR.

Supplementary Table S5. The antibodies and their reaction conditions used in the current study.

Target	KD	Raised in	Application[a]	Dilution	Source	Catalog no.
NRF2	110	Rabbit	Western blot	1:500	Genetex	GTX103322
			Immunofluorescence	1:1000		
			Immunohistochemistry	1:250		
SOX17	55	Goat	ChIP-qPCR	2 ug	R&D	AF1924
			Western blot	1:1000		
		Mouse	Immunohistochemistry	1:100	Origene	CF500096
DNMT1	183	Rabbit	Western blot	1:1000	Genetex	GTX116011
			Immunofluorescence	1:1000		
			Immunohistochemistry	1:500		
DNMT3B	96	mouse	Western blot	1:1000	Abcam	Ab13604
			Immunohistochemistry	1:200		
β-TrCP	-[b]	Rabbit	Immunofluorescence	1:3000	Cell signaling	4394
GPX4	-[b]	Rabbit	Immunohistochemistry	1:500	Abcam	ab41787
4-HNE	-[b]	Rabbit	Immunohistochemistry	1:200	Abcam	ab46545
AMPKα	62	Rabbit	Western blot	1:1000	Cell signaling	5832S
p-AMPKα1/2(T183/T172)	62	Rabbit	Western blot	1:1000	Genetex	GTX63165
mTOR	289	Rabbit	Western blot	1:1000	Cell signaling	2972S
p-mTOR (S2448)	289	Rabbit	Western blot	1:1000	Genetex	GTX79009

GSK-3β	46	Rabbit	Western blot	1:1000	Cell signaling	9315S
p-GSK-3β (Y216)	47	Rabbit	Western blot	1:1000	Abcam	ab75745
GAPDH	37	Mouse	Western blot	1:1000	Santa Cruz	Sc-32233
HDAC1	-[b]	Rabbit	ChIP-qPCR	2 ug	Abcam	ab7028

[a] ChIP-qPCR: Chromatin immunoprecipitation-quantitative polymerase chain reaction

[b] Molecular weight is not applicable to immunohistochemistry or immunofluorescence analysis of this antibody
